# Building energy retrofits in Canada under government fiscal constraints

**DOI:** 10.1038/s41598-026-49147-1

**Published:** 2026-04-27

**Authors:** Ali Madadizadeh, Kamran Siddiqui, Amir A. Aliabadi

**Affiliations:** 1 Department of Mechanical Engineering, University of Guelph, Guelph, ON Canada; 2 Department of Mechanical and Materials Engineering, Western University, London, ON Canada

**Keywords:** Building decarbonization, Climate-equity policy, Energy poverty, Multi-objective optimization, Residential building energy retrofit strategies, Climate sciences, Energy and society, Energy science and technology, Engineering, Environmental sciences

## Abstract

Residential building energy retrofits can significantly reduce greenhouse gas (GHG) emissions and household energy costs when supported by effective fiscal policies. This study develops an integrated simulation–optimization framework to identify optimal retrofit strategies and fiscal parameters across ten Canadian cities. Results indicate that photovoltaic (PV) systems are favored in regions with high grid emission intensity or electricity prices, while thermal insulation levels remain near code minimums and improved air tightness is consistently preferred. Thermal energy storage and heat pumps are effective across most climates. Over a 20-year horizon, these strategies yield up to $7,000 in annual homeowner cost savings and over 100 tonne CO$$_{2}$$e of emissions reductions per building. Financial incentives including rebates ($23,000–$42,000), low-interest loans ($1,600–$8,000), and moderate energy taxes (0.4–8.8%) are essential to ensure affordability and adoption.

## Introduction

The building sector is a critical component of global decarbonization strategies, accounting for nearly one-third of total energy consumption and a substantial share of Greenhouse Gas (GHG) emissions^1–3^. In many developed countries, including Canada, residential buildings are among the most energy-intensive segments due to the aging building stock, which often has poor insulation and inefficient heating/cooling systems. Improving the energy performance of these buildings through retrofitting is widely recognized as one of the most effective and immediate pathways to reduce emissions, improve indoor comfort, and advance national net-zero targets. However, the widespread adoption of energy retrofits remains constrained by high capital costs, limited financial incentives, and uneven access to funding across income groups^4,5^.

To overcome these barriers, governments have introduced a variety of incentive programs such as rebates, low-interest loans, and tax credits. These financial instruments aim to reduce upfront investment costs and encourage homeowners to undertake energy retrofits^6–21^. Yet, most existing programs are designed using static or uniform incentive structures that do not account for variations in climate conditions, building typologies, or socioeconomic diversity. As a result, the effectiveness of such programs often remains suboptimal. In particular, uniform policies may benefit higher-income households with greater financial capacity while failing to reach low- and moderate-income groups who experience higher energy cost burdens and are more vulnerable to energy poverty. Energy poverty, defined as the inability to afford adequate energy services for home heating, cooling, lighting, and appliances, is a growing but under-addressed issue in Canada^22^. More than 8% of Canadian households are classified as energy poor, who spend at least 10% of the household income to cover their energy costs, with energy poor households exceeding 30% in some indigenous and rural communities^23,24^. Achieving an equitable and cost-effective decarbonization pathway, therefore, requires a quantitative understanding of how different policy instruments interact with building parameters, energy performance, and household economics^25^.

Retrofitting the residential building sector is inherently complex because it depends on the interaction of multiple technical, economic, and policy variables^26,27^. At the household level, the extent to which a building can be retrofitted is strongly constrained by the homeowner’s financial capacity and willingness to invest in upgrades that improve energy performance. At the same time, government expenditures play a decisive role: public investment levels vary across regions, and the choice of financial instruments such as grants, rebates, low-interest loans, or targeted taxes directly shapes retrofit feasibility and adoption. Among these instruments, energy taxes remain one of the most debated because they influence both sides of the equation: they can generate revenue that helps governments recover the costs of rebate or loan programs, while simultaneously encouraging homeowners to pursue retrofits to avoid higher energy bills^28,29^. Determining the appropriate tax level is therefore critical; too low offers little incentive, while too high can burden households without yielding proportional retrofitting benefits. Ultimately, society’s investment in retrofitting must be evaluated not only in financial terms but also through its environmental benefits. Significant variation exists across regions in the magnitude of operational and embodied GHG emissions savings achievable through energy retrofit pathways, and identifying which combinations of economic policies and building upgrades deliver the greatest environmental benefit is essential for designing effective and equitable decarbonization strategies.

Optimization methods offer powerful tools to address this challenge by identifying the most efficient combinations of energy retrofit measures and incentive policies under realistic constraints. Previous studies have primarily focused on optimizing building design or technology selection to minimize energy use and emissions^30–36^. However, relatively few have integrated financial policy design within the optimization framework, particularly under fiscal limitations that reflect government budget constraints. This omission limits the ability of policymakers to evaluate trade-offs between public investment, private savings, and environmental benefits. Addressing this gap requires a comprehensive approach that simultaneously considers technological, economic, and policy dimensions of the retrofit decision-making process.

### Research gap and objectives

Despite extensive research on building energy retrofit optimization, existing studies predominantly focus on minimizing energy consumption, lifecycle cost, or emissions from a homeowner perspective, often treating policy instruments as external or fixed parameters. Consequently, there remains a critical gap in integrating government fiscal mechanisms such as rebates, low-interest loans, and energy taxation directly into the optimization process, particularly across diverse climatic and economic conditions.

To address this gap, this study develops a multi-objective simulation-optimization framework that simultaneously evaluates building retrofit strategies and fiscal policy instruments under varying energy price inflation rates. The primary objective is to identify cost-effective, low-carbon retrofit solutions that balance homeowner affordability, government expenditure, and both operational and embodied GHG emissions.

The novelty of this work lies in embedding fiscal instruments directly within the optimization structure, enabling the co-optimization of technical retrofit measures and policy parameters. This integrated approach moves beyond conventional technology-focused analyses by providing a policy-responsive decision-making framework that captures real world economic constraints. Using a representative Canadian housing archetype across multiple climate zones, the framework quantifies the complex interactions among incentive levels, energy price escalation, household cost savings, and emissions reductions, offering new insights into region specific retrofit strategies and their socioeconomic and environmental trade-offs. To guide the analysis, the study addresses the following research questions: (1) How do optimal retrofit strategies vary across Canadian cities under different climate zones and energy price inflation rates? (2) What levels of financial support are required from homeowners and governments to enable adoption? (3) How effective are policy instruments in influencing retrofit decisions and reducing costs? (4) What are the impacts on GHG emissions? (5) To what extent can energy poverty be alleviated? (6) What trade-offs arise among economic and environmental objectives?

## Methodology

**Fig. 1:**
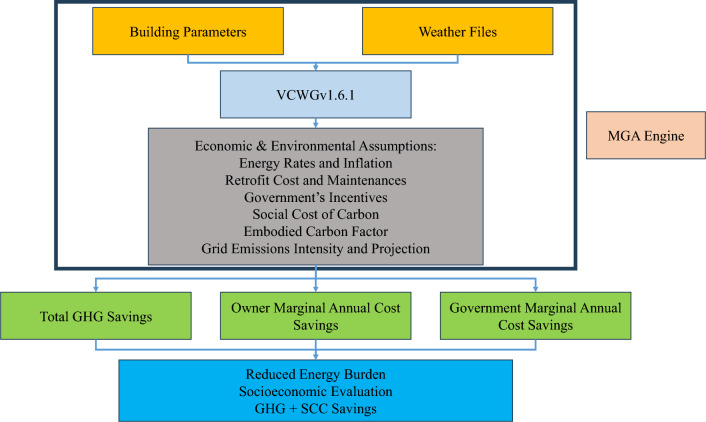
Flowchart for methodological framework.

Figure 1 presents the overall methodological framework adopted in this study. This study focuses on single-detached residential houses in Canada, which account for 52.6% of residential building stock in the country^37^, while other building archetypes can also be studied in future work. City specific weather files and detailed building parameters serve as inputs to the Vertical City Weather Generator (VCWGv1.6.1), an urban physics model that enables high resolution (hourly) simulation of building energy performance under diverse Canadian climates. Full details of this model are provided in our previous publication^5^. In addition to these physical inputs, a range of economic and environmental variables are incorporated into the analysis, including energy price inflation rates (low at 1% annually and high at 5% annually), retrofit and maintenance costs, government incentives, Social Cost of Carbon (SCC), embodied GHG emissions factors, and current and projected electricity grid emission intensities. A Micro-Genetic Algorithm (MGA) is employed to perform a multi-objective optimization of 14 key building retrofit and economic parameters. The objective functions are designed to simultaneously maximize GHG emission savings, maximize homeowners’ marginal annualized cost savings, and maximize governments’ marginal annualized cost savings. The optimal retrofit strategies identified through this process are then evaluated in terms of their broader socioeconomic and environmental impacts, particularly with respect to reducing household energy burdens and supporting equitable decarbonization across Canadian communities with regard to the fiscal constraints.

### Vertical City Weather Generator (VCWGv1.6.1)

**Fig. 2:**
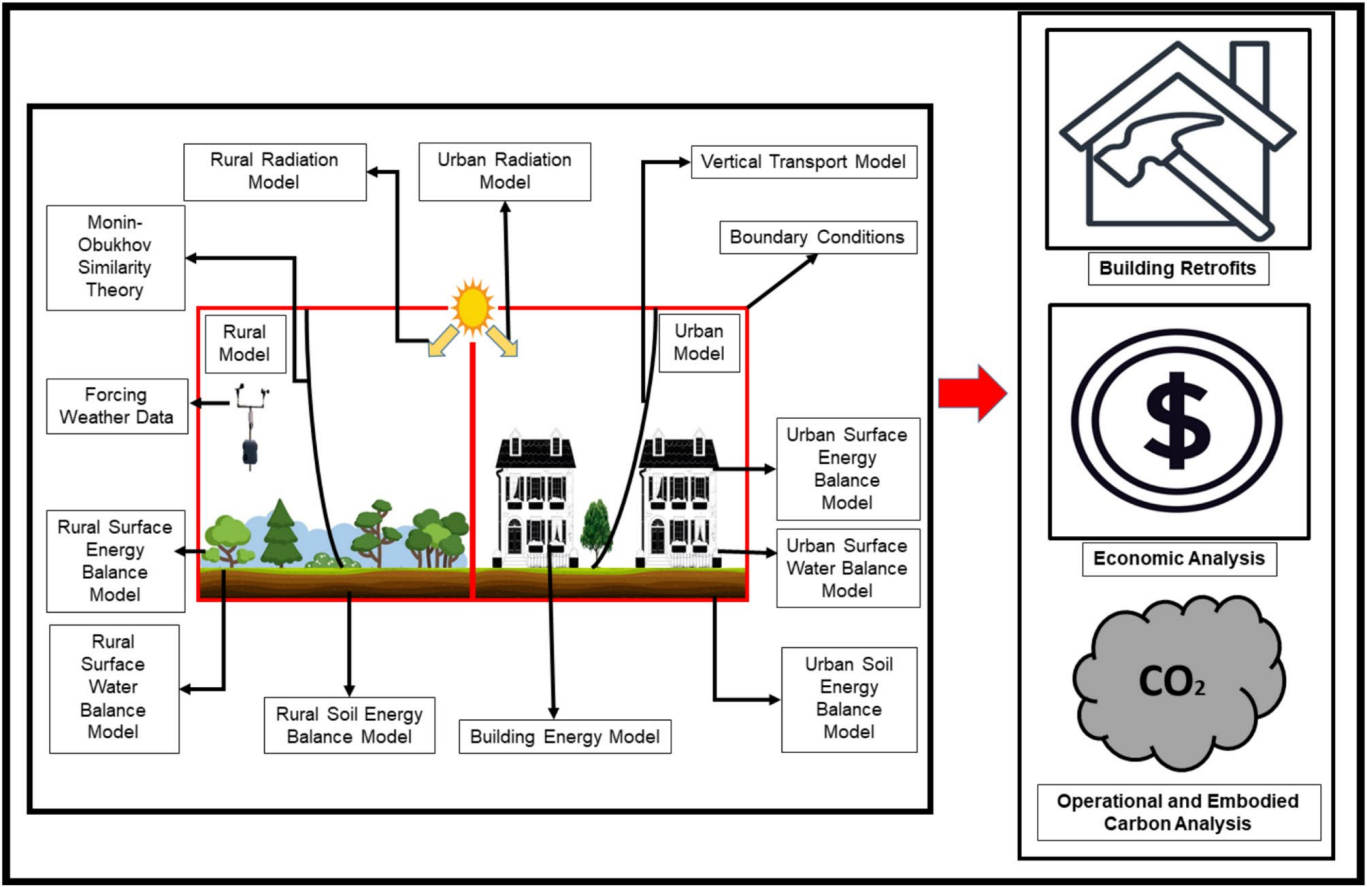
Vertical City Weather Generator (VCWGv1.6.1).

In this study, the Vertical City Weather Generator (VCWG v1.6.1) is employed as an urban physics model to simulate the energy performance and carbon emissions of residential buildings across 10 Canadian cities spanning diverse climate zones. These cities include Vancouver (Zone 4), Toronto (Zone 5), Montreal (Zone 6), Halifax (Zone 6), St. John’s (Zone 6), Calgary (Zone 7A), Winnipeg (Zone 7A), Saskatoon (Zone 7A), Whitehorse (Zone 7B), and Yellowknife (Zone 8)^5,38^. VCWG integrates localized weather data and detailed building parameters for hourly simulations of building energy performance under varying retrofit scenarios. City-specific weather files for the year 2020, capturing hourly air temperature, wind, air pressure, air humidity, radiation fluxes, and soil moisture/temperature, are used as boundary conditions^39^. Each simulation compares baseline (pre-retrofit) and post-retrofit building performance, accounting for interventions using high thermal insulation and air tightness for the envelope, roof albedo modification, Building Integrated Thermal Energy Storage (BITES) system, Heat Pump (HP) system (ground source), Solar Thermal (ST) collector, PhotoVoltaic (PV) collector, Glazing Ratio (GR) adjustments, and Solar Heat Gain Coefficient (*SHGC*) adjustments^40^. The HP performance is modeled using a temperature-dependent Coefficient Of Performance (COP) formulation derived from Natural Resources Canada (NRCan) guidelines^41^ and implemented within the established VCWG framework^40^. The COP varies as a function of heat source and sink temperatures, allowing realistic representation of seasonal changes. The BITES system is modeled as a sensible thermal energy storage medium that stores/releases thermal energy. Charging and discharging occurs through ground, ST collectors, HP operation, and heat recovery from building systems (e.g. ventilation exhaust and water drain). The governing energy balance equations follow the formulation presented in our previous study^40^, ensuring thermodynamic consistency between storage, HP operation, and building load dynamics. The model calculates both operational GHG emissions, derived from electricity and fossil fuel consumption, and embodied GHG emissions associated with retrofit materials and construction. The VCWG framework includes modular sub-models that simulate atmospheric transport, surface energy balances, radiation exchange, and building energy dynamics^42–45^. It also supports the incorporation of key economic and environmental parameters including local energy prices^46–62^, forecasts for energy price inflation rates (1% and 5%), retrofit and maintenance costs, government incentives scenarios (rebates, loans), interest rates for loans payment, taxes for energy consumption, electricity grid emission intensities and projections^63,64^, and the SCC^65^ to provide a comprehensive basis for retrofit analysis^5^. The SCC is incorporated in this study as a policy-relevant economic parameter representing the monetized societal damages associated with greenhouse gas emissions. Although the SCC is not directly paid by homeowners as a separate line item, it ultimately manifests as a private cost through multiple mechanisms. Carbon pricing systems, fuel taxes, regulatory compliance costs, insurance premiums associated with climate-related risks (e.g., flooding and wildfire), and public expenditures financed through taxation all transmit climate damage costs to households over time^66,67^. Therefore, including the SCC allows the optimization framework to internalize long-term climate externalities in a manner consistent with real-world policy instruments. Energy price inflation rates are modeled as forward-looking scenario assumptions reflecting plausible market trajectories rather than fixed policy commitments, enabling evaluation of retrofit performance under varying future economic conditions. The model’s outputs have been validated through comparisons with real-world measurements of environmental and building performance variables from cities such as London (Canada), Guelph (Canada), Vancouver (Canada), and Basel (Switzerland), confirming its suitability for simulating urban building performance variables across varied climatic and urban contexts^4,42,68^ (Fig. 2).

### Micro-genetic algorithm (MGA)

**Fig. 3:**
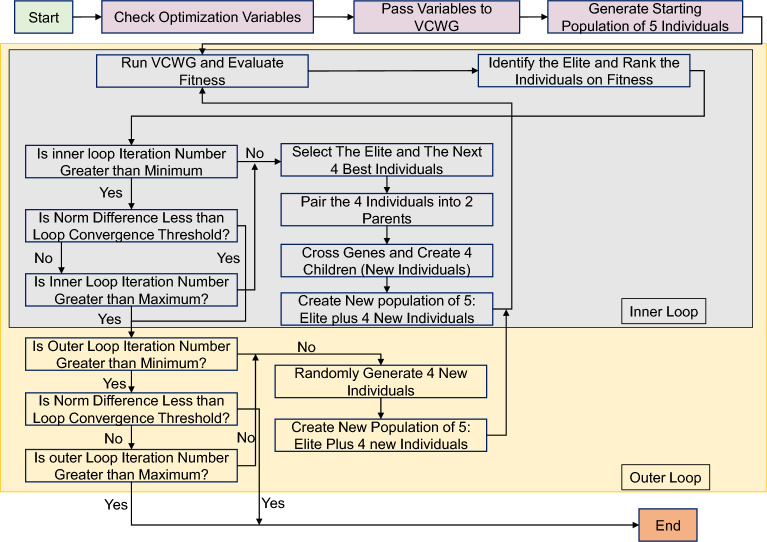
Micro-genetic algorithm (MGA).

The Micro-Genetic Algorithm (MGA) is employed to search for optimal retrofit solutions^4^. MGA is a computationally efficient version of traditional genetic algorithms, designed to solve problems involving multiple conflicting objectives. The algorithm operates by evolving a population of potential solutions (population of 5) (representing different retrofit and government incentive strategies) over a series of generations (up to 100), selecting the best individuals based on the lowest fitness according to the defined objective functions (Fig. 3). The primary goal of this study is to identify retrofit strategies that maximize the homeowner’s annualized marginal cost saving (C$_{Os}$), the government’s annualized marginal cost saving (C$_{Gs}$), and life cycle GHG emissions savings (GHG$_{s}$) over $N=20$ years of each retrofit strategy (Eqn. 1)^4,69^. These objectives are considered simultaneously in the optimization process using a weighted sum approach, which is to be minimized

1 $$\begin{aligned} F = -w_{GHG} \frac{\text {GHG}_{s}}{\text {GHG}_{0s}} - w_{O} \frac{C_{Os}}{C_{0s}} - w_{G} \frac{C_{Gs}}{C_{0s}}. \end{aligned}$$

A sensitivity analysis on the weighting factors was conducted to evaluate the robustness of the results. Because the primary objective of this study is to improve homeowner affordability while achieving decarbonization, a higher weight was assigned to annual marginal homeowner cost savings (0.6), with lower but balanced weights assigned to life-cycle GHG emission savings (0.2) and annual government cost savings (0.2). The detailed results of the weighting sensitivity analysis are provided in the Supplementary Table S2. So we assign specific weights to each sub-objective function, such that $$w_{GHG} = w_{G} = 0.2$$ and $$w_{O} = 0.6$$. The sub-objective functions are normalized using $$\text {GHG}_{0s}$$ [kg-CO$$_2$$e] and $$C_{0s} = |C_{0Os}|+|C_{0Gs}|$$ [$], representing the total GHG emissions savings and the overall absolute value of annualized cost savings, respectively. These values are derived from the solution of the first iteration of the optimization process.

Using the multi-objective optimization framework, we determine the nearly global optimum values for 14 key retrofit variables (outlined in Table 1) under 4 projected energy price inflation rate scenarios.

**Table 1:** Optimization variables with minimum value, maximum value, and variation interval: Building Integrated Thermal Energy Storage (BITES), Solar Thermal (ST), PhotoVoltaic (PV); note: the ranges are informed by building codes and standards in each climate zone^70–72^.

Category	Variables	Minimum	Maximum	Interval
**Building Parameters**	Volume of BITES ($$V_\text {bites}$$) [m$$^{3}$$m$$^{-2}$$]	0.05	0.25	0.04
	Roof Albedo ($$\alpha _R$$)	0.1	0.7	0.05
	Collector Area for ST ($$A_\text {st}$$) [m$$^{2}$$m$$^{-2}$$]	0.2	0.6	0.05
	Roof Thermal Resistance ($$R_\text {roof}$$) [m$$^{2}$$K W$$^{-1}$$]	4.41–7.04	9–14	0.5
	Infiltration Rate ($$V_\text {inf}$$) [ACH]	0.5	1.5	0.25
	Wall Thermal Resistance ($$R_\text {wall}$$) [m$$^{2}$$K W$$^{-1}$$]	3.18–5.46	6.5–11	0.5
	Glazing Ratio (*GR*)	0.1	0.4	0.05
	Solar Heat Gain Coefficient (*SHGC*)	0.1	0.7	0.1
	Collector Area for PV ($$A_\text {pv}$$) [m$$^{2}$$m$$^{-2}$$]	0.1	0.6	0.1
**Economic Parameters**	Rebate [$]	20000	50000	5000
	Loan [$]	0	10000	2000
	Loan Interest Rate [%]	0.25	1.5	0.25
	Fuel Tax [%]	0	10	2
	Electricity Tax [%]	0	10	2

### Economic analysis

The equations in this section are adapted from^4,5^. For the retrofitted case, the annualized cost for the homeowner is given by

2 $$\begin{aligned} C_{\text {Retrofit}} = C_I + C_F + C_E + C_{OM} + L + \text {Tax}_{F} + \text {Tax}_{E} - \text {Rebate} - \text {Loan} - C_S - \text {SCC}_S, \end{aligned}$$

where $$C_I$$ is the annualized initial investment, $$C_F$$ is the annualized fossil fuel cost, $$C_E$$ is the annualized grid electricity consumption cost, $$C_{OM}$$ is the annualized operation and maintenance cost, *L* is the annualized loan cost, $$\text {Tax}_{F}$$ is tax that homeowners pay for their fuel consumption, $$\text {Tax}_{E}$$ is tax that homeowners pay for electricity consumption, $$C_S$$ is the annualized cost savings by salvaging the equipment, and SCC$$_S$$ is annualized cost savings due to reduction of the social cost of carbon, all in [$]. The marginal annualized cost saving is given by

3 $$\begin{aligned} C_{Os} = C_{\text {Base}} - C_{\text {Retrofit}}, \end{aligned}$$

where $$C_{\text {Base}} = C_F + C_E + C_{OM} + \text {Tax}_{F} + \text {Tax}_{E} - C_S$$ is the annualized cost for the base case without any retrofits in [$]. More detailed calculations of the marginal annualized cost are provided in our recently published paper^5^. The government’s annualized marginal cost saving is calculated as follows:

4 $$\begin{aligned} C_{Gs} = L + \text {Tax}_{F} + \text {Tax}_{E} - \text {Loan} - \text {Rebate}. \end{aligned}$$

Supplementary Table S1 indicates the parameters for economic evaluation of energy retrofit strategies. In this study, electricity pricing is modeled under a net metering framework, where households can buy and sell electricity at the same retail rate. Some provinces (e.g. Ontario) utilize the Time Of Use (TOU) scheme, while others have step structures to charge for grid electricity consumption. Regarding fossil fuel consumption, most provinces have fixed rates or step structures for consumption. VCWG considers all of these mechanisms in the economic analysis^4,5^.

### Environmental analysis

The environmental analysis involves the calculation of potential operational GHG emissions savings (GHG$_{o,s}$) and the potential embodied GHG emissions savings (GHG$_{e,s}$), which will be negative in our analysis. The embodied GHG emission, for various building retrofit technologies, is the summation of embodied GHG emissions for each retrofit technology

5 $$\begin{aligned} \begin{aligned} \text {GHG}_{e} =&\ A_{\text {pv}} \text {EEF}_{\text {pv}} + V_{\text {BITES}} \text {EEF}_{\text {BITES}} + \text {EEF}_{\text {HP}} \\&+ A_{\text {st}} \text {EEF}_{\text {st}} + \Delta R_\text {wall} A_{\text {wall}} \text {EEF}_{\text {Insulation}} \\&+ \Delta R_\text {roof} A_{\text {roof}} \text {EEF}_{\text {Insulation}} + A_{\text {roof}} \text {EEF}_{\text {CoolRoof}} \end{aligned} \end{aligned}$$

where $A_{\text {pv}}$, $A_{\text {st}}$, $A_{\text {wall}}$, and $A_{\text {roof}}$, are PV, ST, wall, and roof areas, all in [$\text {m}^2$], $V_{\text {BITES}}$ is the BITES volume in [$\text {m}^3$]; embodied GHG emissions factor (EEF) for PV systems ($\text {EEF}_{\text {pv}}$) is 150 $\text {kg CO}_2\text {e} \text {m}^{-2}$, for the BITES system ($\text {EEF}_{\text {BITES}}$) is 30 $\text {kg CO}_2\text {e} \text {m}^{-3}$, for Heat Pump (HP) $\text {EEF}_{\text {HP}}$ is 600 $\text {kg CO}_2\text {e}$, and for solar thermal systems ($\text {EEF}_{\text {st}}$) is 40 $\text {kg CO}_2\text {e} \text {m}^{-2}$. Additionally, the embodied GHG emissions factor for insulation materials ($\text {EEF}_{\text {Insulation}}$) is 10 $\text {kg CO}_2\text {e} \text {m}^{-2} \text {W} \text {m}^{-2} \text {K}^{-1}$. Here $\Delta R_\text {wall}$ and $\Delta R_\text {roof}$ [m$^{2}$K W$^{-1}$] are changes made to the building envelop to achieve a new thermal resistance. For the cool roof, the embodied GHG emissions factor ($\text {EEF}_{\text {CoolRoof}}$) is 5 $\text {kg CO}_2\text {e} \text {m}^{-2}$^73–76^.

The potential operational GHG emissions saving is the summation of GHG emissions saving through a reduction of electricity and fossil fuel consumption. The fuel usage reduction is computed as

6 $$\begin{aligned} F_s = [F_{hB} + F_{whB} - (F_{h} + F_{wh})] A_{\text {bld}}N, \end{aligned}$$

where $F_{hB}$, $F_{whB}$, $F_{h}$ and $F_{wh}$ [m$^3$ m$^{-2}$] are the fossil fuel usage for the base and retrofitted buildings for space and water heating, respectively. Here $N=20$ is the number of years for the retrofit time horizon. $A_{\text {bld}}$ = 130 [$\text {m}^2$] is the building footprint area. Then, the GHG emissions reduction potential in CO$_2$e associated with fossil fuel saving is estimated by,

7 $$\begin{aligned} \mathrm{GHG}_{F,s} = F_s \rho _{F} \frac{MW_{{\text {CO}_{2}}}}{MW_{F}}, \end{aligned}$$

where $\rho _{F}$ [kg$_F$ m$^{-3}$] is the density of fossil fuel (for natural gas or diesel), $MW_{\text {CO}_2}$ [g$_{\text {CO}_2}$ mole$^{-1}$] is the molecular weight of CO$_2$, and $MW_{F}$ [g$_{F}$ mole$^{-1}$] is the molecular weight of the fossil fuel. The electricity usage reduction is computed by,

8 $$\begin{aligned} E_s = [E_{cB} + E_{dB} - (E_{c} + E_{h} + E_{d} - E_{pv})] A_{\text {bld}}N, \end{aligned}$$

where $E_{cB}$, $E_{dB}$, $E_{c}$, $E_{h}$, and $E_{d}$ [kW-hr m$^{-2}$] are the electricity usage for space cooling/heating and domestic appliance in base/retrofitted buildings, and $E_{pv}$ [kW-hr m$^{-2}$] is electricity generated by PV, in the retrofitted building. Then, the GHG emissions reduction potential in CO$_2$e associated with grid electricity saving is found as

9 $$\begin{aligned} \mathrm{GHG}_{E,s} = E_s EI_{E} P_{E}, \end{aligned}$$

where $EI_{E}$ [kg$_{\text {CO}_2}$ kW-hr$^{-1}$] presents the electricity grid emissions intensity on an annual basis for different cities in 2020, and $P_{E}$ [%] denotes the projected percentage reduction in this intensity over the next 20 years^63,64^. Finally, the potential operational GHG emissions savings is given by

10 $$\begin{aligned} \mathrm{GHG}_{o,s} = \mathrm{GHG}_{F,s} + \mathrm{GHG}_{E,s}, \end{aligned}$$

and the total GHG emission savings over $N=20$ years is given by:

11 $$\begin{aligned} \mathrm{GHG}_{s} = \mathrm{GHG}_{o,s} - \mathrm{GHG}_{e}. \end{aligned}$$

## Results and discussion

The results of this study present a comprehensive assessment of how optimized retrofit strategies influence household energy performance, economic outcomes, and environmental impacts across the ten Canadian cities. First, we examine the optimization outcomes and associated trade-offs, focusing on the resulting homeowner cost savings, government investment, and GHG emissions reductions, followed by a detailed analysis of the optimized economic variables and building parameters that drive these outcomes. Next, we quantify the extent to which retrofits alleviate household energy burden, highlighting differences among regions and retrofit configurations. Finally, we evaluate the broader environmental and economic co-benefits of building decarbonization, demonstrating how well-designed incentive structures and retrofitting measures contribute to long-term sustainability and affordability objectives.

### Optimization outcomes and trade-offs

This section summarizes the outcomes of the multi-objective optimization by examining how different retrofit strategies balance environmental benefits with economic considerations for both households and the government. The optimization scheme simultaneously maximizes three key objectives: total GHG emissions savings over a 20-year horizon, annual marginal homeowner cost savings, and annual marginal government cost savings. Because these objectives compete, measures that yield high long-term GHG reductions may impose higher upfront or ongoing costs, the results highlight important trade-offs that influence retrofit adoption and policy design. The following subsections present the optimized levels of cost and emissions savings, identify the economic variables selected by the model, and describe the optimal building parameters that emerge across different climate regions and future energy price inflation rate scenarios.

#### Cost and GHG emissions savings

The convergence behavior of the optimization scheme was consistent across all cities. Supplementary Fig. S1 provides an example of the normalized overall objective function convergence trends for Toronto across different runs with varying energy price inflation rates. The overall objective function value showed a clear downward trend over successive generations of the MGA. This decreasing trend eventually stabilized, indicating that the search space was sufficiently explored and the algorithm had converged toward a global or near-global optimum. The final sub-objective function values were nearly constant across the last few iterations, confirming the robustness of the optimization framework and the stability of the solutions. Although MGA does not guarantee to find the global optimum solution^4^, this convergence pattern was observed consistently across all cities and scenarios, providing confidence in the validity and transferability of the optimized retrofit strategies.

**Fig. 4:**
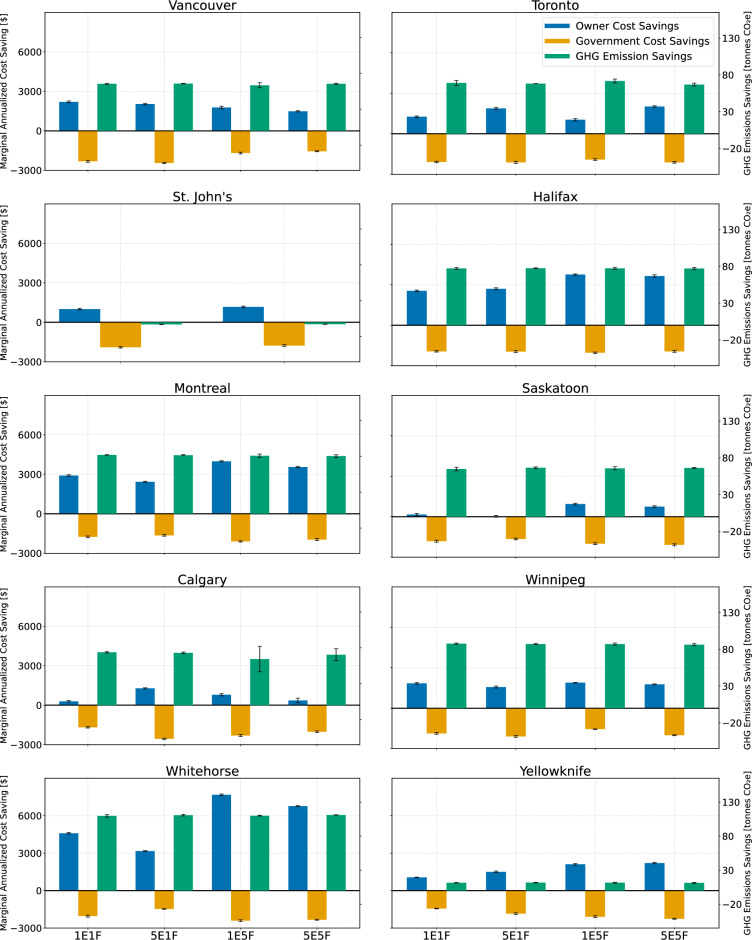
Sub-objective functions for different cities under different energy price inflation rates scenarios; legend: blue: homeowners’ marginal annual cost savings, orange: government marginal annual cost savings, green: GHG emissions savings (20 years).

Across the ten Canadian cities analyzed, the combined results, shown in Fig. 4, reveal consistent patterns in how retrofit outcomes respond to variations in electricity and fuel price inflation rates. In all locations, homeowners’ marginal annual cost savings remain positive or near-positive in most scenarios, indicating that energy retrofits generally reduce operational energy expenditures for households. In Vancouver, homeowners’ savings are actually higher under lower energy price inflation rates. This occurs because electricity is relatively inexpensive in British Columbia, and the city’s availability of shortwave solar radiation limits the economic return from rooftop PV installations, making retrofit savings less sensitive to rising prices. In Toronto, however, the opposite pattern emerges: homeowners’ savings increase with a higher electricity price inflation rate but decrease when fuel price inflation rate rises. This is due to the high electricity prices in Toronto and the strong financial benefit of net-metered rooftop PV, which allows households to generate and sell electricity.

In St. John’s, where households rely heavily on electricity for both heating and cooling, the retrofit benefits are modest but remain generally positive due to reduced electricity consumption combined with government rebates or loans. Halifax and Montreal show clear financial gains for the homeowners from retrofits across all scenarios, with homeowners’ savings increasing as both electricity and fuel price inflation rates rise. Conversely, in Saskatoon and Calgary, homeowners’ cost savings remain relatively small, although still positive, owing to lower baseline energy prices. Winnipeg exhibits a stable pattern in which homeowners’ savings remain fairly consistent across all energy price inflation rate scenarios.

The northern territories demonstrate the strongest financial response to fuel price inflation rate. In Whitehorse, retrofit measures yield substantial savings for the homeowners, and the benefits grow sharply under higher diesel fuel price inflation rate. This is mainly due to the dominant heating demand and the significant reduction in fuel consumption achieved by deep retrofit measures, including HP adoption (ground source). Yellowknife shows a similar but slightly less pronounced trend.

As shown in Fig. 4, government’s marginal annual cost savings are negative in nearly all cities and under every energy price inflation rate scenario, clearly demonstrating that achieving low-carbon buildings and reducing household energy burden cannot occur without meaningful public investment. This directly answers a central policy question: Is it possible to realize lower-carbon housing while reducing energy poverty without government spending? The results indicate that the answer is unequivocally ‘no’. According to our analysis, local, provincial, territorial, and federal governments must provide financial support through rebates, loans, or tax-based instruments if deep retrofits are to be adopted at scale and remain affordable for households. Across all cities, governments’ cost savings remain negative because incentives and subsidies consistently exceed any fiscal returns from reduced energy consumption or increased tax revenue. Moreover, the magnitude of negative savings tends to increase slightly when energy price inflation rates rise, implying that communities require even greater government support in scenarios of high energy costs. This reflects the economic reality that, as energy prices escalate, larger incentives are needed to keep retrofit investments financially viable for homeowners and to ensure that retrofits continue to deliver meaningful reductions in household energy cost burden. Overall, these results highlight that decarbonizing the building stock and alleviating energy poverty are not cost-neutral processes for governments; rather, they require sustained and adaptive fiscal commitment, especially under volatile or rising energy prices.

The total GHG emissions savings (Fig. 4), combining both operational and embodied carbon emissions, are positive across most cities and energy price inflation rate scenarios, confirming that retrofit packages deliver meaningful environmental benefits in most regions. The largest reductions occur in cold-climate cities with carbon-intensive heating fuels, where electrification and the deployment of BITES, HP, and ST systems substantially reduce heating loads and shift energy demand toward lower-carbon electricity. In cities such as Vancouver, Montreal, and Winnipeg, which already operate on relatively low grid emission intensities due to hydro-dominated electricity, GHG emissions reductions primarily arise from replacing natural gas heating with cleaner technologies. These cities benefit less from fuel switching alone, but still achieve significant savings through load reduction and improved system efficiencies. In Toronto, where electricity prices are high but the grid is low-carbon intensity, GHG emissions savings come from two pathways: (1) reducing natural gas consumption through HP, BITES, and ST technologies, and (2) generating on-site renewable electricity via rooftop PV. Net-metering further results in GHG emissions savings by offsetting grid electricity with clean on-site generation.

In Halifax, Saskatoon, and Calgary, where grid emission intensities remain higher, the optimization framework prioritizes reducing natural gas use while simultaneously increasing PV deployment to offset carbon-intensive electricity. This dual mechanism helps achieve significant GHG emissions reductions even in regions with comparatively high-carbon intensity grids. A notable exception is St. John’s, where GHG emissions savings are slightly negative. This occurs because households already rely predominantly on electricity for both heating and cooling, and the provincial grid is largely green. As a result, retrofitting does not meaningfully reduce GHG emissions, even though it may still improve energy affordability for households.

In the northern territories, particularly Whitehorse, the highest GHG emissions reductions are observed. The combination of extremely high heating demand, carbon-intensive fuel use (primarily diesel), and high energy price inflation rates makes retrofit technologies highly impactful. As energy prices rise, the optimized retrofit strategies shift more aggressively toward electrification and deep efficiency improvements, amplifying GHG emissions savings potential even further.

#### Optimized economic parameters

Optimized rebate allocations, representing direct government contributions toward retrofit costs, do not vary widely across different cities. In Vancouver rebates span from $25,000 under the 5E5F scenario to $40,000 under the 5E1F scenario. Toronto exhibits a similar pattern, with rebates clustering around $34,000 in most energy price inflation rate scenarios. The only notable deviation occurs under 1E5F, where the rebate decreases to $31,000 because high fuel price inflation rate independently improves the financial case for electrification-oriented retrofits, reducing the level of government support needed. Across most of the remaining cities including Calgary, Halifax, Montreal, Saskatoon, Winnipeg, Whitehorse, and Yellowknife, rebate levels generally fall within a narrower band around $30,000 (see Table 2 and Supplementary Fig. S8-17).

Loan allocations and interest rates also adjust dynamically to local climatic and economic conditions. In Vancouver, loan amounts increase from $2,800 in the 1E1F scenario to $6,000 under 5E5F, reflecting the need for higher upfront financing as energy price inflation rates magnify the economic value of retrofit measures. Optimized interest rates for loans remain very low (0.55-0.95%), indicating that the optimization framework relies on low-interest financing to preserve affordability for homeowners while still achieving deep GHG emission reductions. Toronto shows a similar moderate loan range ($2,800–$7,200) with interest rates around 0.85–1.10%, again highlighting the importance of accessible financing for large, electricity-driven retrofit packages. In several other cities including Halifax, Saskatoon, Calgary, Winnipeg, Whitehorse, Yellowknife, and St. John’s, the pattern is reversed: loan amounts tend to decrease as electricity and fuel price inflation rates increase. In these regions, higher energy price escalation already strengthens the economic case for retrofits, reducing the need for large loans. Across nearly all cities, interest rates remain below 1%, typically fluctuating only slightly with energy price inflation rate assumptions, indicating that the model consistently favors low-interest public financing as an effective policy lever to stimulate retrofit adoption (see Table 2 and Supplementary Fig. S8-17).

Another key economic parameter optimized in Table 2 is the energy consumption tax applied to electricity and fossil fuel usage. Setting this tax appropriately is critical: a tax that is too high can increase the energy cost burden for households, while a tax that is too low provides little incentive for homeowners to conserve energy or invest in energy retrofits. Across most Canadian cities, the model identifies a declining tax requirement as electricity and fuel price inflation rates increase. For instance, in Vancouver, the optimal electricity tax decreases from 6% to 4%, and the fuel tax drops slightly from 4.0% to 3.6% as energy price inflation rates rise from 1E1F to 5E5F, indicating that higher energy prices already motivate households to reduce consumption thus lowering the need for additional policy pressure through taxation. Toronto shows an even more pronounced decline, with electricity tax falling from 8% to 4% and fuel tax from 6.4% to 4% as energy price inflation rates rise from 1E1F to 5E5F. A similar downward trend is observed in St. John’s (7.2% to 3.6%), Montreal, Saskatoon, and Calgary, demonstrating that in these regions, the increasing energy price inflation rates strengthen the economic incentive for retrofits, reducing the need for taxation as a behavioral tool. In contrast, cities such as Winnipeg, Whitehorse, and Yellowknife exhibit the opposite pattern, where energy taxes increase under higher energy price inflation rates scenarios. These colder regions rely heavily on fuel-based heating, and the model suggests that when energy price inflation rates rise, a higher energy tax is needed to encourage retrofits and reduce fuel consumption. Overall, the optimization indicates that energy consumption taxes must be regionally differentiated.

**Table 2:** Optimized economic parameters for each city under different electricity (E) and fuel (F) price inflation rate (percent) scenarios (average ± standard deviation over 5 runs).

City	Scenarios	Rebate [$]	Loan [$]	Loan Interest Rate [%]	Electricity Tax [%]	Fuel Tax [%]
**Vancouver**	1E1F	38000 ± 13038	2800 ± 3033	0.95 ± 0.41	6.00 ± 4.00	4.00 ± 2.45
	5E1F	40000 ± 7906	4000 ± 3162	0.55 ± 0.41	5.60 ± 3.58	4.40 ± 2.19
	1E5F	27000 ± 10368	5200 ± 4147	0.70 ± 0.41	7.20 ± 4.38	4.40 ± 3.85
	5E5F	25000 ± 5000	6000 ± 3742	0.95 ± 0.21	4.40 ± 3.85	3.60 ± 2.61
**Toronto**	1E1F	34000 ± 8215	3200 ± 3633	1.00 ± 0.47	8.00 ± 2.00	6.40 ± 2.61
	5E1F	34000 ± 12942	3200 ± 2280	0.85 ± 0.14	6.00 ± 1.41	3.60 ± 2.97
	1E5F	31000 ± 9617	2800 ± 3346	1.10 ± 0.45	4.40 ± 4.34	8.80 ± 1.79
	5E5F	34000 ± 9617	7200 ± 2683	0.85 ± 0.29	4.00 ± 4.24	4.00 ± 2.83
**St. John’s**	1E	31000 ± 9620	6000 ± 2449	0.70 ± 0.54	7.20 ± 2.28	-
	5E	29000 ± 10840	4400 ± 3578	0.90 ± 0.51	3.60 ± 4.10	-
**Halifax**	1E1F	31000 ± 9618	5600 ± 3286	1.00 ± 0.31	6.80 ± 3.63	2.40 ± 3.29
	5E1F	29000 ± 12450	4800 ± 3033	0.40 ± 0.22	2.80 ± 2.68	8.80 ± 2.68
	1E5F	31000 ± 8216	4800 ± 3633	0.85 ± 0.38	1.60 ± 1.67	5.20 ± 3.63
	5E5F	30000 ± 11726	4800 ± 3633	0.95 ± 0.37	6.40 ± 3.85	3.20 ± 2.68
**Montreal**	1E1F	29000 ± 10839	3200 ± 4604	1.25 ± 0.18	6.80 ± 3.90	3.20 ± 2.28
	5E1F	27000 ± 10368	2000 ± 2828	0.90 ± 0.38	5.20 ± 3.03	4.00 ± 3.74
	1E5F	33000 ± 9082	3200 ± 4147	1.25 ± 0.18	5.20 ± 3.03	6.40 ± 4.98
	5E5F	31000 ± 11937	7200 ± 4381	0.90 ± 0.38	4.40 ± 2.61	4.80 ± 5.02
**Saskatoon**	1E1F	30000 ± 12747	5600 ± 3577	1.05 ± 0.62	6.80 ± 2.28	4.40 ± 1.67
	5E1F	27000 ± 10954	4800 ± 4816	0.55 ± 0.27	5.20 ± 5.02	4.80 ± 3.63
	1E5F	33000 ± 11510	4400 ± 2190	0.95 ± 0.41	7.20 ± 1.79	3.60 ± 2.19
	5E5F	34000 ± 11937	4000 ± 2828	0.70 ± 0.33	5.60 ± 4.10	5.60 ± 3.85
**Calgary**	1E1F	27000 ± 8366	6800 ± 4147	0.80 ± 0.44	6.00 ± 4.24	6.40 ± 4.09
	5E1F	42000 ± 9082	5200 ± 4147	1.00 ± 0.30	5.20 ± 2.28	6.40 ± 2.19
	1E5F	38000 ± 12549	2400 ± 2190	0.90 ± 0.57	4.40 ± 4.56	4.40 ± 3.57
	5E5F	33000 ± 10954	3600 ± 3286	0.65 ± 0.33	4.80 ± 2.68	4.00 ± 2.82
**Winnipeg**	1E1F	31000 ± 9618	6000 ± 2449	0.45 ± 0.32	4.80 ± 5.02	3.60 ± 4.33
	5E1F	35000 ± 11180	3200 ± 4147	0.60 ± 0.33	3.60 ± 4.33	5.20 ± 3.03
	1E5F	25000 ± 3536	4000 ± 2449	0.75 ± 0.35	2.40 ± 2.19	7.20 ± 3.89
	5E5F	34000 ± 5477	3200 ± 3033	0.90 ± 0.28	7.20 ± 3.03	5.20 ± 4.14
**Whitehorse**	1E1F	32000 ± 13038	4800 ± 5019	0.50 ± 0.25	2.40 ± 1.67	2.80 ± 1.79
	5E1F	23000 ± 2738	4000 ± 4690	0.65 ± 0.38	4.00 ± 2.83	3.60 ± 2.97
	1E5F	35000 ± 11726	8000 ± 1414	1.10 ± 0.58	8.00 ± 3.46	5.60 ± 2.61
	5E5F	35000 ± 6123	2800 ± 4147	0.80 ± 0.37	4.00 ± 4.24	4.40 ± 3.58
**Yellowknife**	1E1F	23000 ± 4472	6000 ± 1414	0.45 ± 0.20	4.00 ± 3.46	4.00 ± 3.74
	5E1F	30000 ± 11726	2800 ± 1788	1.30 ± 0.20	0.40 ± 0.89	8.00 ± 2.44
	1E5F	34000 ± 11937	3200 ± 2280	1.05 ± 0.37	5.60 ± 3.28	5.20 ± 3.63
	5E5F	37000 ± 7582	1600 ± 2190	0.95 ± 0.41	7.20 ± 4.38	4.80 ± 4.14

#### Optimized building parameters

From the optimized building parameters perspective, as shown in Table 3, the model selects retrofit strategies that reflect each city’s local energy costs, climate conditions, and grid emission intensities. In Vancouver (also see Supplementary Fig. S18), which has a mild climate (Zone = 4), frequent cloud cover, green electricity, and relatively low electricity prices, the optimization consistently selects a minimal rooftop PV area (0.10–0.14 m$^{2}$m$^{-2}$). Because insulation upgrades offer limited economic returns under these conditions, both roof and wall *R*-values remain near their minimum code-based limits, with only minor variations across economic scenarios. The infiltration rate decreases from 1.5 ACH toward approximately 1.0 ACH, indicating that tightening the building envelope provides an optimal balance between maintaining sufficient natural ventilation and reducing heating/cooling demands. The *SHGC* is pushed to its maximum allowable value ($\sim$0.70), reflecting the benefit of passive solar heat gains in Vancouver’s climate. Meanwhile, the glazing ratio stays in a moderate range (around 0.20), suggesting that the model limits glazing to avoid excessive heat loss/gain while still permitting passive daylighting. Importantly, the optimization identifies BITES, ST, and HP as key retrofit components for Vancouver. Because the city’s electricity supply is already low-carbon intensity, shifting heating from natural gas to electrified systems supplemented by thermal storage yields meaningful GHG emissions reductions while keeping operational costs low. Roof albedo values remain in the mid-range ($\sim$0.4), indicating that a moderately reflective roof provides year-round benefits helping reduce summer cooling loads without imposing excessive winter heating penalties (see Table 3).

In Toronto, the optimized retrofit solutions consistently favor higher PV deployment, reflecting the city’s relatively high electricity prices and favorable direct shortwave solar radiation availability. Because envelope upgrades (roof and wall *R*-values) provide limited economic returns under Ontario’s incentive structure, the model keeps these parameters near the minimum bounds defined by building codes and standards. The *SHGC* is pushed to its maximum value, enhancing passive solar heat gains during Toronto’s cold winters, while the glazing ratio remains in a moderate range to balance daylighting against heat loss/gain penalties. The optimized infiltration rate stabilizes around 1.0–1.2 ACH, indicating envelope tightening without over-restricting natural ventilation. The model also selects high levels of BITES, mid-range ST deployment, and HP (ground source), technologies that support electrification and reduce natural gas consumption. Notably, ST area increases from 0.38 to 0.48 m$^{2}$m$^{-2}$ as energy price inflation rates rise from 1E1F to 5E5F, demonstrating how higher future energy costs make solar thermal heating increasingly cost-effective. These selections reflect the combined influence of Ontario’s relatively higher grid electricity GHG intensity, colder winters, and a high electricity price, where a coordinated package of PV, ST, HP, and thermal storage delivers substantial reductions in both GHG emissions and long-term homeowners’ energy costs (see Table 3 , Supplementary Fig. S19).

In Halifax, the optimized retrofit configurations show moderate but consistent improvements across different energy price inflation rate scenarios. Roof insulation remains close to the code-minimum range (5.56–5.76 m$^{2}$K W$^{-1}$), and wall insulation stays within 4.05–4.25 m$^{2}$K W$^{-1}$, confirming that additional envelope insulation provides limited economic benefit relative to other measures. In contrast, PV deployment is consistently high at 0.6 m$^{2}$m$^{-2}$, reflecting Halifax’s favorable solar potential and high grid emission intensity, which increases the GHG emissions reduction value of on-site electricity generation. The model strongly favors BITES, selecting relatively high storage volumes across all scenarios, which indicates that thermal storage plays a meaningful role in reducing heating demand and stabilizing energy use in Halifax’s cold and humid climate. Infiltration rates range from 0.85 to 1.10 ACH, suggesting that moderate envelope tightening is cost-effective, especially when fuel prices rise and heating energy becomes more expensive. The *SHGC* is consistently at the upper bound ($\sim$0.70), showing that passive solar heat gains are beneficial in Halifax’s climate to offset heating demand during colder seasons. Meanwhile, the glazing ratio remains in the mid-range, balancing daylighting benefits with the need to control heat loss/gain (see Table 3, Supplementary Fig. S20).

Given Montreal’s extremely low-carbon intensity and low-cost electricity supply, electrification becomes the most effective pathway. HPs (ground source) address heating needs well, while BITES provides thermal storage that reduces peak heating demand, and ST captures useful solar heat during winters. This integrated approach allows the building to decrease fuel consumption without relying heavily on costly envelope retrofits. Because electricity in Montreal is both clean and inexpensive, PV deployment is not favored by the optimization (0.10 m$^{2}$m$^{-2}$) across different economic scenarios. PV provides limited economic benefit compared to other technologies in this region. Meanwhile, the glazing ratio and *SHGC* vary within the mid-to-high range, allowing the model to optimize passive solar heat gains in winter while keeping summer cooling loads manageable across different energy price inflation rate scenarios. Overall, Montreal’s optimal retrofit strategy leverages province’s clean electricity grid and focuses on thermal storage, solar thermal gains, and electrification, rather than envelope upgrades or large-scale PV installations (see Table 3, Supplementary Fig. S21).

St. John’s exhibits the smallest retrofit interventions overall. The optimized BITES volume is very low (0.066–0.082 m$^{3}$m$^{-2}$), indicating that added thermal inertia provides minimal benefit in Newfoundland’s climatic and economic context. The roof and wall *R*-values remain fixed at near-minimum values, and ST area drops from 0.34 to 0.22 m$^{2}$m$^{-2}$ when electricity price inflation rate increases from 1E to 5E. PV area remains minimal (0.10 m$^{2}$m$^{-2}$), as St. John’s has low solar potential. Infiltration remains mid range for the optimized solution (see Table 3, Supplementary Fig. S22).

In Calgary, the optimization results identify retrofit strategies that rely heavily on solar-driven technologies while maintaining envelope characteristics close to code-minimum levels. Although Calgary experiences long and cold winters, the *R*-values for both roof and wall insulation remain near the lower bound of the optimization space, confirming that increasing thermal insulation does not provide strong economic returns relative to other retrofit measures. The model allocates substantial retrofit effort to ST and PV, both of which are highly effective in Calgary due to the province’s strong solar resource and high grid GHG emissions intensity. Solar thermal area varies considerably across scenarios (0.20–0.42 m$^{2}$m$^{-2}$), reflecting the model’s attempt to balance solar heat gains with fuel displacement under different electricity and fuel price inflation rates. Similarly, PV deployment remains consistently favored (0.54–0.60 m$^{2}$m$^{-2}$), indicating that on-site clean electricity generation is economically and environmentally attractive in Alberta’s carbon-intensive grid. Optimized BITES volumes show significant variability (0.10–0.25 m$^{3}$m$^{-2}$), suggesting that thermal storage is selectively valuable under scenarios where fuel prices or heating loads intensify. The glazing ratio also varies across energy price inflation rate scenarios, allowing the model to increase passive solar heat gain in colder conditions while controlling overheating risks in milder periods (see Table 3, Supplementary Fig. S23).

Saskatoon demonstrates strong reliance on operational strategies such as solar thermal and PV (always 0.6 m$^{2}$m$^{-2}$). Despite extremely cold winters, the optimization maintains roof and wall *R*-values near the lower code limits (6.17–6.47 m$^{2}$K W$^{-1}$ for roof and 4.76–4.96 m$^{2}$K W$^{-1}$ for wall), because additional insulation provides diminishing returns beyond the regulatory minimum. Instead, the optimization adjusts the albedo (0.22–0.44) and BITES volume (0.19–0.23 m$^{3}$m$^{-2}$) to manage peak loads and improve long-term economic outcomes (see Table 3, Supplementary Fig. S24).

In Winnipeg, the optimization results indicate stable yet relatively modest retrofit interventions, influenced by the city’s cold climate but also its low-carbon intensity electricity supply. Roof and wall insulation values remain close to the code-minimums (approximately 6.27 m$^{2}$K W$^{-1}$ for roofs and 4.76 m$^{2}$K W$^{-1}$ for walls), confirming that added insulation does not generate sufficient economic return to justify higher material costs within the optimization framework. Because Manitoba’s electricity grid is already highly renewable and low in carbon intensity, the model selects minimal PV deployment (0.10–0.12 m$^{2}$m$^{-2}$). In such a clean grid, PV provides limited GHG emissions reduction benefits, and its economic attractiveness is weaker than in provinces with higher emission intensities. Conversely, ST is selected at moderate levels (0.26–0.34 m$^{2}$m$^{-2}$), helping offset space-heating demand during Winnipeg’s long winters. Variables such as *SHGC* remain at their maximum value, enabling greater passive solar heat gains in a cold climate. The optimized glazing ratio is $\sim$0.3, balancing daylighting and solar gain while avoiding excessive heat loss. The optimization also prioritizes BITES and HP, which complement Manitoba’s clean electricity by shifting heating loads from fossil fuels to low-carbon intensity electricity. These measures offer environmental benefits without requiring large capital investments in envelope upgrades (see Table 3, Supplementary Fig. S25).

Whitehorse, in the far north, shows moderate optimized BITES volumes (0.23–0.25 m$^{3}$m$^{-2}$) but maintains low PV levels (0.10–0.14 m$^{2}$m$^{-2}$) due to limited winter solar availability. *R*-values again remain near the minimum code requirement, reinforcing that insulation amount beyond code does not significantly improve the trade-off function in extremely cold climates where heating loads dominate regardless. The optimized solar thermal collector area is moderate (0.36–0.46 m$^{2}$m$^{-2}$), and infiltration is maintained around 0.90–1.05 ACH. Yellowknife exhibits the lowest optimized BITES volumes (0.058–0.066 m$^{3}$m$^{-2}$), which shows that large thermal inertia does not improve energy performance in this extreme subarctic climate. *R*-values remain fixed near the minimum code requirement (around 7.0 m$^{2}$K W$^{-1}$ for roof and 5.5 m$^{2}$K W$^{-1}$ for wall). Optimized solar thermal collector area is moderate (0.20–0.44 m$^{2}$m$^{-2}$), while glazing ratios remain relatively low. The optimized infiltration rate is high compared to other cities (around 1.15–1.30 ACH), indicating that envelope tightening was not prioritized by the optimization engine. PV deployment remains very small (0.10–0.12 m$^{2}$m$^{-2}$) owing to limited solar resource and low marginal benefit (see Table 3, Supplementary Figs. S26-S27).

**Table 3:** Optimized building parameters for each city under different electricity (E) and fuel (F) price inflation rate (percent) scenarios (average ± standard deviation over 5 runs).

City	Scenarios	$V_\text {bites}$ [m$^{3}$m$^{-2}$]	$\alpha _R$	$A_\text {st}$ [m$^{2}$m$^{-2}$]	$R_\text {roof}$ [m$^{2}$K W$^{-1}$]	$R_\text {wall}$ [m$^{2}$K W$^{-1}$]	*GR*	$V_\text {inf}$ [ACH]	*SHGC*	$A_\text {pv}$ [m$^{2}$m$^{-2}$]
**Vancouver**	1E1F	0.234 ± 0.035	0.560 ± 0.114	0.420 ± 0.178	4.610 ± 0.273	3.180 ± 0.000	0.190 ± 0.124	1.100 ± 0.418	0.700 ± 0.000	0.100 ± 0.000
	5E1F	0.242 ± 0.017	0.440 ± 0.251	0.380 ± 0.178	4.510 ± 0.223	3.180 ± 0.000	0.210 ± 0.114	0.900 ± 0.379	0.700 ± 0.000	0.100 ± 0.000
	1E5F	0.202 ± 0.086	0.320 ± 0.204	0.300 ± 0.187	4.710 ± 0.447	3.280 ± 0.223	0.270 ± 0.057	1.050 ± 0.512	0.680 ± 0.044	0.140 ± 0.089
	5E5F	0.250 ± 0.000	0.420 ± 0.258	0.300 ± 0.158	4.610 ± 0.447	3.280 ± 0.223	0.250 ± 0.141	1.100 ± 0.379	0.700 ± 0.044	0.100 ± 0.000
**Toronto**	1E1F	0.242 ± 0.018	0.520 ± 0.268	0.380 ± 0.259	5.460 ± 0.000	3.700 ± 0.224	0.200 ± 0.117	0.950 ± 0.411	0.700 ± 0.000	0.500 ± 0.224
	5E1F	0.250 ± 0.000	0.440 ± 0.207	0.340 ± 0.152	5.460 ± 0.000	3.600 ± 0.000	0.240 ± 0.102	1.100 ± 0.418	0.700 ± 0.000	0.600 ± 0.000
	1E5F	0.250 ± 0.000	0.420 ± 0.179	0.460 ± 0.207	5.660 ± 0.447	3.700 ± 0.224	0.270 ± 0.057	1.200 ± 0.447	0.700 ± 0.000	0.300 ± 0.235
	5E5F	0.250 ± 0.000	0.300 ± 0.212	0.480 ± 0.130	5.460 ± 0.000	3.600 ± 0.000	0.260 ± 0.147	0.950 ± 0.326	0.660 ± 0.055	0.580 ± 0.045
**St. John’s**	1E	0.066 ± 0.022	0.500 ± 0.173	0.340 ± 0.230	5.560 ± 0.224	4.050 ± 0.000	0.270 ± 0.091	1.050 ± 0.512	0.640 ± 0.089	0.100 ± 0.000
	5E	0.082 ± 0.052	0.420 ± 0.192	0.220 ± 0.217	5.460 ± 0.000	4.050 ± 0.000	0.250 ± 0.122	0.900 ± 0.379	0.700 ± 0.000	0.100 ± 0.000
**Halifax**	1E1F	0.242 ± 0.018	0.400 ± 0.224	0.260 ± 0.152	5.660 ± 0.274	4.050 ± 0.000	0.280 ± 0.120	0.850 ± 0.379	0.680 ± 0.045	0.600 ± 0.000
	5E1F	0.234 ± 0.036	0.360 ± 0.152	0.360 ± 0.195	5.660 ± 0.274	4.150 ± 0.224	0.220 ± 0.130	1.050 ± 0.326	0.700 ± 0.000	0.600 ± 0.000
	1E5F	0.234 ± 0.036	0.600 ± 0.100	0.360 ± 0.167	5.560 ± 0.224	4.050 ± 0.000	0.300 ± 0.071	1.100 ± 0.548	0.680 ± 0.045	0.600 ± 0.000
	5E5F	0.162 ± 0.104	0.420 ± 0.192	0.340 ± 0.207	5.760 ± 0.671	4.250 ± 0.274	0.250 ± 0.117	0.950 ± 0.447	0.700 ± 0.000	0.600 ± 0.000
**Montreal**	1E1F	0.242 ± 0.018	0.580 ± 0.130	0.480 ± 0.130	5.560 ± 0.224	4.050 ± 0.000	0.170 ± 0.057	1.100 ± 0.379	0.700 ± 0.000	0.100 ± 0.000
	5E1F	0.250 ± 0.000	0.380 ± 0.259	0.380 ± 0.217	5.560 ± 0.224	4.250 ± 0.274	0.320 ± 0.091	1.000 ± 0.395	0.700 ± 0.000	0.100 ± 0.000
	1E5F	0.242 ± 0.018	0.420 ± 0.217	0.400 ± 0.100	5.560 ± 0.224	4.150 ± 0.224	0.250 ± 0.127	1.200 ± 0.274	0.680 ± 0.045	0.100 ± 0.000
	5E5F	0.226 ± 0.036	0.400 ± 0.071	0.420 ± 0.148	5.460 ± 0.000	4.250 ± 0.274	0.270 ± 0.067	0.900 ± 0.224	0.680 ± 0.045	0.100 ± 0.000
**Saskatoon**	1E1F	0.234 ± 0.022	0.440 ± 0.195	0.280 ± 0.192	6.470 ± 0.447	4.960 ± 0.274	0.290 ± 0.108	0.800 ± 0.209	0.660 ± 0.055	0.600 ± 0.000
	5E1F	0.194 ± 0.088	0.220 ± 0.084	0.280 ± 0.217	6.270 ± 0.224	4.860 ± 0.224	0.270 ± 0.104	1.050 ± 0.209	0.700 ± 0.000	0.600 ± 0.000
	1E5F	0.234 ± 0.022	0.420 ± 0.228	0.200 ± 0.122	6.170 ± 0.000	4.760 ± 0.000	0.260 ± 0.119	0.850 ± 0.418	0.660 ± 0.055	0.600 ± 0.000
	5E5F	0.210 ± 0.069	0.280 ± 0.164	0.320 ± 0.164	6.270 ± 0.224	4.960 ± 0.274	0.280 ± 0.115	1.100 ± 0.285	0.700 ± 0.000	0.600 ± 0.000
**Calgary**	1E1F	0.242 ± 0.018	0.380 ± 0.164	0.420 ± 0.164	6.370 ± 0.274	4.860 ± 0.224	0.300 ± 0.079	1.150 ± 0.418	0.700 ± 0.000	0.600 ± 0.000
	5E1F	0.106 ± 0.088	0.480 ± 0.130	0.360 ± 0.207	6.370 ± 0.274	4.860 ± 0.224	0.160 ± 0.065	0.700 ± 0.209	0.700 ± 0.000	0.600 ± 0.000
	1E5F	0.250 ± 0.000	0.520 ± 0.249	0.200 ± 0.122	6.670 ± 0.707	4.960 ± 0.274	0.190 ± 0.102	1.150 ± 0.379	0.640 ± 0.089	0.540 ± 0.134
	5E5F	0.194 ± 0.088	0.400 ± 0.122	0.340 ± 0.182	6.770 ± 1.342	5.460 ± 1.565	0.240 ± 0.108	1.050 ± 0.411	0.680 ± 0.045	0.600 ± 0.000
**Winnipeg**	1E1F	0.234 ± 0.036	0.400 ± 0.187	0.260 ± 0.152	6.270 ± 0.224	4.760 ± 0.000	0.290 ± 0.108	1.000 ± 0.395	0.700 ± 0.000	0.100 ± 0.000
	5E1F	0.242 ± 0.018	0.460 ± 0.251	0.340 ± 0.167	6.270 ± 0.224	4.860 ± 0.224	0.300 ± 0.117	0.900 ± 0.379	0.700 ± 0.000	0.120 ± 0.045
	1E5F	0.202 ± 0.066	0.320 ± 0.179	0.280 ± 0.148	6.170 ± 0.000	4.760 ± 0.000	0.280 ± 0.076	0.750 ± 0.433	0.700 ± 0.000	0.100 ± 0.000
	5E5F	0.202 ± 0.087	0.600 ± 0.173	0.320 ± 0.228	6.370 ± 0.447	4.860 ± 0.224	0.220 ± 0.084	1.000 ± 0.354	0.700 ± 0.000	0.120 ± 0.045
**Whitehorse**	1E1F	0.226 ± 0.036	0.240 ± 0.114	0.400 ± 0.187	6.270 ± 0.224	4.960 ± 0.274	0.280 ± 0.120	0.900 ± 0.335	0.700 ± 0.000	0.120 ± 0.045
	5E1F	0.242 ± 0.018	0.240 ± 0.167	0.360 ± 0.167	6.270 ± 0.224	4.760 ± 0.000	0.250 ± 0.117	1.050 ± 0.371	0.700 ± 0.000	0.140 ± 0.055
	1E5F	0.242 ± 0.018	0.360 ± 0.195	0.460 ± 0.152	6.670 ± 0.612	4.960 ± 0.274	0.300 ± 0.071	1.050 ± 0.411	0.700 ± 0.000	0.100 ± 0.000
	5E5F	0.250 ± 0.000	0.440 ± 0.167	0.400 ± 0.200	6.570 ± 0.224	4.760 ± 0.000	0.290 ± 0.114	1.000 ± 0.354	0.700 ± 0.000	0.100 ± 0.000
**Yellowknife**	1E1F	0.058 ± 0.018	0.360 ± 0.207	0.420 ± 0.130	7.340 ± 0.274	5.460 ± 0.000	0.190 ± 0.082	1.150 ± 0.285	0.700 ± 0.000	0.100 ± 0.000
	5E1F	0.058 ± 0.018	0.300 ± 0.224	0.440 ± 0.134	7.140 ± 0.224	5.460 ± 0.000	0.250 ± 0.094	1.150 ± 0.335	0.700 ± 0.000	0.100 ± 0.000
	1E5F	0.058 ± 0.018	0.360 ± 0.241	0.440 ± 0.114	7.040 ± 0.000	5.560 ± 0.224	0.230 ± 0.097	1.150 ± 0.224	0.700 ± 0.000	0.100 ± 0.000
	5E5F	0.066 ± 0.022	0.320 ± 0.217	0.200 ± 0.100	7.140 ± 0.224	5.460 ± 0.000	0.290 ± 0.125	1.300 ± 0.209	0.700 ± 0.000	0.120 ± 0.045

Beyond city-specific outcomes, several cross-cutting national patterns emerge. First, insulation levels for both roof and walls consistently remain close to code-minimum values across all climates, including cold regions such as Winnipeg, Saskatoon, Calgary, and Yellowknife. This indicates that, within the optimization framework, increasing envelope insulation beyond current standards yields diminishing economic returns relative to other retrofit options. High upfront capital costs combined with already stringent building codes reduce the marginal benefit of further insulation upgrades. Instead, the model systematically prioritizes air tightness, electrification, solar technologies (PV and ST), thermal storage (BITES) and *SHGC*, which provide stronger combined economic and emissions reduction benefits under most energy price and grid emission intensity conditions. Second, PV deployment is favored with high electricity prices or high grid emission intensities, while remaining minimal in provinces with low-carbon intensity grids or low-cost electricity, where heat pumps can be favored (e.g., Montreal, Winnipeg, Vancouver). These consistent patterns highlight that future retrofit policies should focus less on universal envelope thermal insulation mandates and more on regionally-tailored electrification and renewable strategies that reflect local energy systems and price structures.

### Retrofit impact on household energy burden

**Fig. 5:**
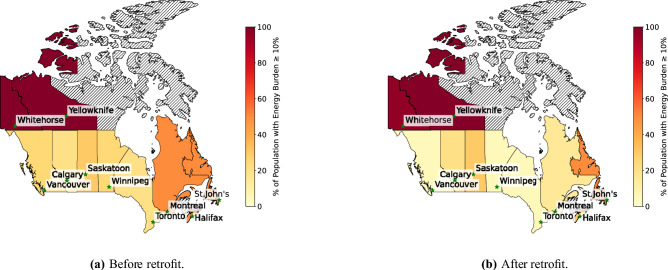
Energy burden comparison for 1E1F energy price inflation rate before and after retrofitting; map generated using python 3.10 and various libraries: geopandas 1.0.1, matplotlib 3.9.0, and unidecode 1.4.0 (https://www.python.org/).

Energy poverty in this study is defined using the widely applied 10% expenditure threshold, whereby a household is considered energy-poor if annual energy costs exceed 10% of gross household income. Household income data were obtained from the Canadian Census published by Statistics Canada, using publicly available city-level income statistics^24,77^. Pre- and post-retrofit annual energy expenditures were calculated based on simulated building energy consumption combined with city-specific utility rates. Baseline energy poverty rates were derived by comparing modeled pre-retrofit energy costs against census-reported household incomes. Post-retrofit energy poverty levels were then recalculated using optimized retrofit energy expenditures under the same income assumptions. This consistent framework ensures that the reported changes in energy poverty are grounded in nationally representative government data and transparent modeling assumptions. The impact of retrofits on energy burden and consequently energy poverty was analyzed for different regions. The results are presented in Fig. 5 (also see Supplementary Figs. S2 to S4). The results show that before retrofits, energy-poverty rates vary substantially across Canadian provinces and territories, reflecting differences in climate conditions, energy sources, and socioeconomic state.

In Vancouver, baseline energy-poverty levels range between 25% and 50% depending on the energy price inflation rate scenario, yet retrofits reduce these burdens to between 5% and 30%, representing one of the most effective socioeconomic improvements nationally. Calgary follows a similar trend, with pre-retrofit levels of 20–30% dropping to 10–20% in most economic scenarios, although the benefits diminish under the highest fuel and electricity price inflation rates (5E5F), indicating stronger sensitivity to combined energy price pressures. Toronto also experiences considerable reductions: pre-retrofit rates of 20–30% fall to 5–10% across different economic scenarios, highlighting how retrofit interventions can substantially ease the energy cost burden for a large share of households in Canada’s most populated city. Montreal, where baseline energy-poverty rates reach 60% under more severe energy price inflation scenarios, shows meaningful decreases after retrofit, with reductions to 15–25%. This represents an improvement and reflects the high thermal loads of Montreal’s building stock and the strong effect of retrofit measures on winter energy demand. The Atlantic provinces exhibit some of the highest baseline energy-poverty burdens in the country. In Halifax, pre-retrofit levels range from 50% to 90%, and although retrofits reduce these to 30–60%, a significant proportion of households continue to face energy affordability challenges. St. John’s trends indicate that while retrofits reduce energy poverty from 50–75% down to 40–75%, the region remains one of the least responsive to retrofit interventions. These findings suggest that while retrofits help, structural challenges such as dependence on electricity-based heating and persistent cold-season demand prevent deeper reductions. The Prairie provinces show moderate but notable improvements. In Calgary, the share of households experiencing energy poverty was approximately 20–30% before retrofitting, and the optimized retrofit strategy reduces this range to roughly 10–25%, indicating a meaningful though moderate improvement in affordability. In Winnipeg, a baseline range of 25–50% contracts to 5–30% after retrofitting, indicating that energy upgrades are highly effective in reducing the energy cost burden for this region. Saskatoon shows a smaller reduction, with energy poverty decreasing from 30–50% to 30–40%. Canada’s northern territories remain the most vulnerable in all scenarios. Both Whitehorse and the Yellowknife cities exhibit near-universal baseline energy poverty (99%). These outcomes highlight the extreme climatic conditions and high operating costs in the North, where even optimized retrofits cannot sufficiently lower energy expenditures to remove households from energy poverty.

Across most of the cities considered, the results demonstrate that retrofits can significantly reduce energy-poverty severity especially in urban southern regions with electricity-based heating while northern and Atlantic regions require additional financial support, energy-price stabilization policies, or fuel-switching measures to achieve comparable gains. Overall, the analysis confirms that optimized retrofit strategies can effectively reduce energy poverty in Canada, but their impact varies strongly with regional energy sources, climate conditions, and energy price inflation pressures. The results reveal significant regional disparities in both baseline energy-poverty levels and the effectiveness of retrofit interventions, underscoring the need for geographically differentiated policy design. In southern urban regions of Ontario, British Columbia, Alberta, and Quebec, retrofits consistently reduce energy poverty by 20-40% across all inflation scenarios. These findings indicate that the proposed incentive structures such as rebates, low-interest loans, and performance-based support can be strategically expanded to accelerate adoption, as the marginal public investment required to lift households out of energy poverty is relatively low compared to the resulting long-term societal and environmental benefits. By contrast, the Atlantic provinces and the northern territories require a substantially different policy approach. Nova Scotia and Newfoundland/Labrador continue to exhibit 50–60% energy-poverty prevalence even after optimized retrofits, suggesting that energy efficiency improvements alone cannot compensate for high energy costs and volatile price exposure. For these regions, retrofit programs must be complemented by targeted measures such as fuel-switching incentives (e.g. to HPs), province-specific energy-price stabilization mechanisms, and expanded low-income energy-support programs. Meanwhile, Yukon and the Northwest Territories demonstrate almost no improvement despite retrofits. This indicates that in the North, extreme climatic conditions, high delivered energy costs, and structural limitations in existing housing stock outweigh the benefits of conventional retrofit packages. Policies for these regions should prioritize large-scale heating system transitions, community energy infrastructure investment, and ongoing subsidies for essential energy services, not just only one time retrofit financial assistance. Importantly, the results also show that the higher electricity and fuel price inflation rate (5E5F scenario) erodes retrofit effectiveness in all provinces, particularly in Alberta, Saskatchewan, and Newfoundland/Labrador. This implies that national strategies to mitigate energy-price volatility through carbon credit redistribution, progressive energy tariffs, or regulated price smoothing may be necessary to ensure that retrofits continue to alleviate, rather than merely buffer, household energy cost burdens under inflationary conditions.

**Fig. 6:**
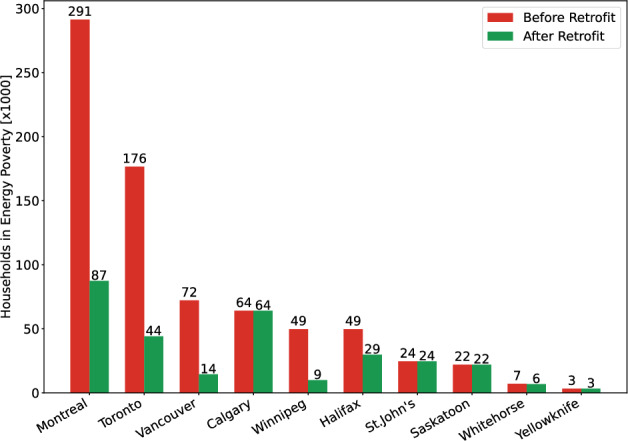
Number of households with energy poverty under 1E1F energy price inflation rate before and after retrofitting.

Finally, because retrofit effectiveness correlates strongly with household count in major cities, directing a larger share of incentives to highly populated cities such as Toronto, Vancouver, and Montreal can maximize national-scale poverty reduction while maintaining equity through enhanced support for high-burden, low-population regions. As shown in Fig. 6, the optimized retrofit strategies under the 1E1F energy price inflation rate scenario can lift approximately 204,000 households in Montreal, 132,000 in Toronto, and 58,000 in Vancouver out of energy poverty. Overall, the findings support a tiered policy architecture combining universal retrofit incentives with region-specific energy affordability interventions to effectively reduce energy poverty across Canada (also see Supplementary Figs. S5 to S7).

### Environmental and economic co-benefits of decarbonization

**Fig. 7:**
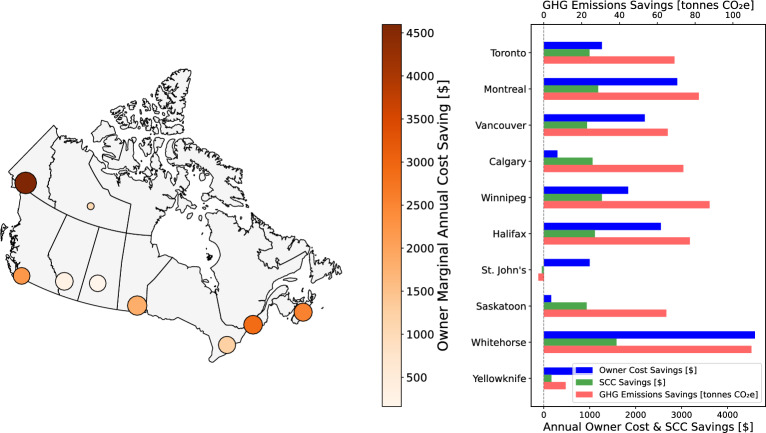
Total GHG emissions savings and annual SCC savings after building energy retrofits under the 1E1F energy price inflation rate. The panel shows GHG emissions savings (size) over a 20-year horizon and annual SCC savings (color); map generated using python 3.10 and various libraries: geopandas 1.0.1, matplotlib 3.9.0, and unidecode 1.4.0 (https://www.python.org/).

The results in preceding sections reveal that retrofitting strategies generate significant GHG emission reductions across all Canadian cities considered in this study, delivering both climate and economic benefits. Cities achieve up to 100 tonnes CO$$_{2}$$e GHG emissions savings over a 20-year time horizon. These GHG emissions reductions correspond to notable Social Cost of Carbon (SCC) savings, estimated using a conservative carbon price benchmark. As shown in Fig. 7, under the 1E1F energy price inflation rate scenario, nearly all cities (except St. John’s) demonstrate robust environmental and economic benefits from optimized retrofit strategies. In Toronto, households achieve approximately $1,000 in SCC savings and reduce GHG emissions by about 70 tonnes CO$$_{2}$$e. Montreal performs even better, with $1,500 in SCC savings and approximately 80 tonnes CO$$_{2}$$e of GHG emissions savings, reflecting the city’s strong alignment with retrofit potential. Calgary also shows significant mitigation benefits, reducing GHG emissions by around 70 tonnes CO$$_{2}$$e, while not too much overall cost savings are possible. Winnipeg exhibits high impact as well, with SCC savings nearing $1,500 and GHG emissions savings of 80 tonnes CO$$_{2}$$e. Notably, Whitehorse stands out as the top-performing city, achieving the highest GHG emissions and SCC savings under these conditions (100 tonnes CO$$_{2}$$e of GHG emissions savings and $1,700 of SCC saving), likely due to its extreme climate, high baseline energy use, and cost of heating fuels. Results for other economic scenarios are featured in Supplementary Figs. S28 to S30. In the 5E1F energy price inflation rate scenario, Whitehorse leads with a reduction of over 100 tonnes CO$$_{2}$$e in GHG emissions, combined with an SCC savings of $1,500, while Toronto achieves over 60 tonnes CO$$_{2}$$e in GHG emissions savings, combined with SCC savings of $1000. GHG emissions savings in cities like Yellowknife and Calgary (approximately 10 and 75 tonnes CO$$_{2}$$e, respectively) demonstrate the nationwide reach of retrofit benefits. Scenarios involving higher fossil fuel energy price inflation rates (1E5F) shift the advantage slightly toward colder regions like Saskatoon, which could save over 65 tonnes CO$$_{2}$$e emissions, while Whitehorse and Winnipeg remain top performers. Toronto continues to benefit from retrofits even under scenarios with higher fossil fuel energy price inflation rates. At a high energy price inflation rate (5E5F), Winnipeg and Halifax both reach 80 tonnes CO$$_{2}$$e of GHG emissions savings, with SCC benefits exceeding $1,000. Montreal results are similar across all economic scenarios, consistently saving over 80 tonnes CO$$_{2}$$e of emissions and over $1,000 in SCC, showing similar results regardless of energy price inflation rate scenarios.

### Boundaries and limitations

This study presents an integrated simulation-optimization framework to evaluate building energy retrofit strategies under fiscal policy constraints. While the framework provides valuable insights, several boundaries and limitations should be acknowledged. First, the analysis is based on a specific representative residential building archetype, which may not fully capture the diversity of building characteristics, occupant behavior, and retrofit feasibility across the housing stock. Variations in construction quality, year of construction, occupancy patterns, and user behavior are not explicitly modeled and may influence actual energy performance and cost savings. Second, the building energy simulations rely on predefined assumptions regarding system performance, operational schedules, and technology specifications. Although these assumptions are consistent with standard practice, real-world performance may deviate due to installation quality, maintenance, and climatic variability. Third, the economic analysis incorporates simplified representations of fiscal policy instruments, including rebates, low-interest loans, and energy taxes. The implementation of such policies in practice may involve administrative constraints, eligibility criteria, and regional variations that are not fully captured in the model. Fourth, future electricity and fuel price trajectories are represented through predefined inflation scenarios. While these scenarios aim to reflect plausible uncertainties, actual energy price evolution may differ due to market dynamics, regulatory changes, technological, and political disruptions.

## Conclusion

This study developed an integrated simulation-optimization framework to identify cost-effective and low-carbon intensity retrofit strategies across ten Canadian cities under varying energy price inflation rates. By coupling building energy modeling with fiscal policy instruments, the framework evaluates trade-offs between homeowner affordability, government expenditure, and GHG emissions reductions. Results show that optimal retrofit strategies are strongly influenced by regional climate conditions, energy prices, and grid emissions intensity. Active technologies, including HPs, BITES, ST systems, and PV systems, consistently outperform envelope improvements, with insulation levels remaining close to code minimums and air tightness improvements and increasing SHGC playing key roles. PV deployment is maximized in regions with high electricity costs or carbon-intensive grids, while it is reduced in low-emission or low-solar-potential regions. From an economic perspective, moderate rebates, low-interest loans, and targeted energy taxes are sufficient to enable widespread retrofit adoption while balancing fiscal constraints. These policy instruments play a critical role in reducing upfront costs and influencing technology selection, highlighting the importance of integrated policy design. The analysis also demonstrates that optimized retrofit strategies can significantly reduce household energy burden and contribute to alleviating energy poverty, although regional disparities persist.

Moreover, these findings emphasize large-scale residential decarbonization and energy poverty reduction cannot be achieved without sustained public investment. Although government marginal savings are negative in most scenarios, these expenditures represent strategic climate and social infrastructure investments rather than short-term fiscal losses. Retrofit programs reduce long-term climate damages, improve household energy affordability, and enhance energy system resilience, generating benefits that extend beyond immediate budget balances. The results indicate that effective policy design requires deliberate prioritization of public resources, balancing fiscal responsibility with long-term environmental and social objectives. Governments face trade-offs similar to other public spending domains such as healthcare, education, housing, and infrastructure, and the scale of investment in building decarbonization, ultimately reflects societal priorities. Overall, the results highlight that large-scale residential decarbonization requires coordinated technological and policy solutions. Well-designed incentive structures can simultaneously reduce emissions, improve affordability, and support long-term climate and social objectives.

## Supplementary Information

Supplementary Information.

## Data Availability

The Atmospheric Innovations Research (AIR) Laboratory at the University of Guelph provides the model source code. For access, contact Amir A. Aliabadi (aaliabad@uoguelph.ca), visit http://www.aaa-scientists.com/, or visit https://github.com/AmirAAliabadi.

## References

[1] Canada Green Building Council. *Building Climate Solutions: The Role of Green Buildings in Addressing Climate Change*. https://www.cagbc.org/why-green-building/building-climate-solutions (2023). Accessed: 2025-09-01.

[2] Efficiency Canada. *Reaching Net Zero in Existing Buildings*. https://www.efficiencycanada.org/building-codes/reaching-net-zero-in-existing-buildings (2023). Accessed: 2025-09-01.

[3] Madadizadeh, A., Siddiqui, K. & Aliabadi, A. A. Review: The economics landscape for building decarbonization. *Sustainability* **16**, 6214. 10.3390/su16146214 (2024).

[4] Aliabadi, A. A., Chen, X., Yang, J., Madadizadeh, A. & Siddiqui, K. Retrofit optimization of building systems for future climates using an urban physics model. *Build. Environ.* **243**, 110655. 10.1016/j.buildenv.2023.110655 (2023).

[5] Madadizadeh, A., Gharabaghi, B., Siddiqui, K. & Aliabadi, A. A. Efficacy of government incentivized residential building retrofits in Canada. *Sci. Rep.* **15**, 26062. 10.1038/s41598-025-10264-y (2025). 40681576 10.1038/s41598-025-10264-y PMC12274604

[6] Natural Resources Canada. *Canada Greener Homes Initiative*. https://natural-resources.canada.ca/energy-efficiency/homes/canada-greener-homes-initiative/24831 (2024). Accessed: 20 March 2024.

[7] City of Toronto. *Green Your Roof Program*. https://www.toronto.ca/services-payments/water-environment/environmental-grants-incentives/green-your-roof/ (2024). Accessed: 20 March 2024.

[8] City of Toronto. *Home Energy Loan Program (Help)*. https://www.toronto.ca/services-payments/water-environment/environmental-grants-incentives/home-energy-loan-program-help/ (2024). Accessed: 20 March 2024.

[9] Better Homes BC. *Better Homes BC Program*. https://www.betterhomesbc.ca/ (2024). Accessed: 20 March 2024.

[10] Clean Energy Improvement Program (CEIP). City of Calgary - Residential ceip. https://ceip.abmunis.ca/residential/locations/city-of-calgary/ (2024). Accessed: 20 March 2024.

[11] Efficiency Nova Scotia. *Residential Programs*. https://www.efficiencyns.ca/residential/ (2024). Accessed: 20 March 2024.

[12] City of Saskatoon. *Home Energy Loan Program*. https://www.saskatoon.ca/environmental-initiatives/energy-water/home-energy-loan-program-help (2024). Accessed: 20 March 2024.

[13] SaskEnergy. *Home Efficiency Retrofit Rebate*. https://www.saskenergy.com/ways-save/home-efficiency-retrofit-rebate (2024). Accessed: 20 March 2024.

[14] Manitoba Hydro. *Home Energy Efficiency Loan*. https://www.hydro.mb.ca/account/loans/home-energy-efficiency-loan/ (2024). Accessed: 20 March 2024.

[15] Efficiency Manitoba. *Solar Program*. https://efficiencymb.ca/solar/ (2024). Accessed: 20 March 2024.

[16] Government of Newfoundland and Labrador. Home energy savings program. https://www.gov.nl.ca/ecc/occ/low-carbon-economy-programs/homeenergysavings/ (2024). Accessed: 20 March 2024.

[17] Take Charge NL. Residential programs. https://takechargenl.ca/residential/ (2024). Accessed: 20 March 2024.

[18] Transition énergétique Québec. Rénoclimat - financial assistance. https://transitionenergetique.gouv.qc.ca/en/residential/programs/renoclimat/financial-assistance (2024). Accessed: 20 March 2024.

[19] Energir. Energy efficiency grants. https://energir.com/en/residential/customer-centre/reduce-my-consumption/energy-efficiency-grants (2024). Accessed: 20 March 2024.

[20] Arctic Energy Alliance. Rebates and services. https://aea.nt.ca/rebates-services/ (2024). Accessed: 20 March 2024.

[21] Government of Yukon. Home energy rebates. https://yukon.ca/en/housing-and-property/home-energy-rebates (2024). Accessed: 20 March 2024.

[22] Herrero, S. T. Energy poverty indicators: A critical review of methods. *Indoor Built Environ.* **26**, 1018–1031. 10.1177/1420326X17718054 (2017).

[23] Tardy, F. & Lee, B. Building related energy poverty in developed countries-past, present, and future from a Canadian perspective. *Energy Build.* **194**, 46–61. 10.1016/j.enbuild.2019.04.013 (2019).

[24] Canada, S. Estimation of energy poverty rates using the 2021 census of population. https://www150.statcan.gc.ca/n1/pub/46-28-0001/2024001/article/00001-eng.htm (2024). Accessed: 2025-08-02.

[25] Madadizadeh, A., Siddiqui, K. & Aliabadi, A. A. Building retrofits and economic analysis across canada: Natural ventilation and photovoltaic systems. In *CSME/CFD2024* (Eindhoven, 2024).

[26] Bertoldi, P., Economidou, M., Palermo, V., Boza-Kiss, B. & Todeschi, V. How to finance energy renovation of residential buildings: Review of current and emerging financing instruments in the eu. *Wiley Interdiscip. Rev.* **10**, e384. 10.1002/wene.384 (2021).

[27] Madadizadeh, A., Siddiqui, K. & Aliabadi, A. A. A Multi-objective Optimization Algorithm For Building Systems Retrofits. In *the 6th International Conference on Building Energy and Environment (COBEE 2025)* (Eindhoven, 2025).

[28] Okwandu, A. C., Esho, A. O.-O., Iluyomade, T. D. & Olatunde, T. The role of policy and regulation in promoting green buildings. *World J. Adv. Res. Rev.* **22**, 139–150, 10.30574/wjarr.2024.22.1.1047 (2024).

[29] Tamasiga, P., Onyeaka, H., Bakwena, M. & Ouassou, E. H. Pricing the future: Unveiling the effects of carbon pricing on socio-economic outcomes and energy poverty. *Int. J. Sustain. Energy* **43**, 2362334. 10.1080/14786451.2024.2362334 (2024).

[30] Wetter, M. & Wright, J. A comparison of deterministic and probabilistic optimization algorithms for nonsmooth simulation-based optimization. *Build. Environ.* **39**, 989–999. 10.1016/j.buildenv.2004.01.022 (2004).

[31] Kämpf, J. H., Wetter, M. & Robinson, D. A comparison of global optimization algorithms with standard benchmark functions and real-world applications using EnergyPlus. *J. Build. Perform. Simul.* **3**, 103–120. 10.1080/19401490903494597 (2010).

[32] Tuhus-Dubrow, D. & Krarti, M. Genetic-algorithm based approach to optimize building envelope design for residential buildings. *Build. Environ.* **45**, 1574–1581. 10.1016/j.buildenv.2010.01.005 (2010).

[33] Bamdad, K., Cholette, M. E., Guan, L. & Bell, J. Building energy optimisation under uncertainty using ACOMV algorithm. *Energy Build.* **167**, 322–333. 10.1016/j.enbuild.2018.02.053 (2018).

[34] Wang, J., Chen, H., Yuan, Y. & Huang, Y. A novel efficient optimization algorithm for parameter estimation of building thermal dynamic models. *Build. Environ.* **153**, 233–240. 10.1016/j.buildenv.2019.02.006 (2019).

[35] Bamdad, K., Cholette, M. E., Omrani, S. & Bell, J. Future energy-optimised buildings – addressing the impact of climate change on buildings. *Energy Build.* **231**, 110610. 10.1016/j.enbuild.2020.110610 (2021).

[36] Mukkavaara, J. & Shadram, F. An integrated optimization and sensitivity analysis approach to support the life cycle energy trade-off in building design. *Energy Build.* **253**, 111529. 10.1016/j.enbuild.2021.111529 (2021).

[37] NRCan. National Energy Use Database. Tech. Rep., Office of Energy Efficiency, Natural Resources Canada, Gatineau (2020).

[38] ASHRAE. Standard 169: Climatic Data for Building Design Standards. Tech. Rep., American Society for Heating Refrigeration and Airconditioning Engineers, Peachtree Corners (2020).

[39] Aliabadi, A. A. & McLeod, R. M. The Vatic Weather File Generator (VWFG v1.0.0). *J. Build. Eng.* **67**, 105966. 10.1016/j.jobe.2023.105966 (2023).

[40] Aliabadi, A. A., Moradi, M., McLeod, R. M., Calder, D. & Dernovsek, R. How much building renewable energy is enough? The Vertical City Weather Generator (VCWG v1.4.4). *Atmosphere* **12**, 882, 10.3390/atmos12070882 (2021).

[41] NRCan. Heating and Cooling with a Heat Pump. Tech. Rep., Office of Energy Efficiency, Natural Resources Canada, Gatineau, QC (2004).

[42] Moradi, M. *et al.* The Vertical City Weather Generator (VCWG v1.3.2). *Geosci. Model Dev.* **14**, 961–984, 10.5194/gmd-14-961-2021 (2021).

[43] Moradi, M. *The Vertical City Weather Generator*. Ph.D. thesis, University of Guelph, Guelph (2021).

[44] Aliabadi, A. A. *Turbulence: A Fundamental Approach for Scientists and Engineers* (Springer, 2022).

[45] Safdari, M., Janaideh, M. A., Siddiqui, K. & Aliabadi, A. A. Weather-adaptive fuzzy control of setpoints for energy-efficient hvac in urban buildings. *J. Build. Eng.* **104**, 112317. 10.1016/j.jobe.2025.112317 (2025).

[46] Ontario Energy Board. Electricity rates. https://www.oeb.ca/consumer-information-and-protection/electricity-rates (2023). Accessed: 3 December 2023.

[47] Utilities Consumer Advocate. Regulated rates. https://ucahelps.alberta.ca/regulated-rates.aspx (2023). Accessed: 3 December 2023.

[48] BC Hydro. Residential rates. https://app.bchydro.com/accounts-billing/rates-energy-use/electricity-rates/residential-rates.html (2023). Accessed: 3 December 2023.

[49] Manitoba Hydro. Residential rates. https://www.hydro.mb.ca/account/billing/rates/residential/ (2023). Accessed: 3 December 2023.

[50] SaskPower. Residential billing information. https://www.saskpower.com/accounts/billing/your-power-bill/how-to-read-your-bill/residential (2023). Accessed: 3 December 2023.

[51] Nova Scotia Power. Domestic service tariff. https://www.nspower.ca/about-us/producing/rates-tariffs/domestic-service-tariff (2023). Accessed: 3 December 2023.

[52] Newfoundland Power. Electricity rates. https://www.newfoundlandpower.com/My-Account/Usage/Electricity-Rates (2023). Accessed: 3 December 2023.

[53] Hydro-Québec. Residential rates. https://www.hydroquebec.com/residential/customer-space/rates/ (2023). Accessed: 3 December 2023.

[54] NAKAPower. Rates and regulations. https://www.nakapower.com/en-ca/customer-billing-rates/rates-regulations.html (2023). Accessed: 3 December 2023.

[55] ATCO Electric Yukon. Bill calculator and rates. https://www.atcoelectricyukon.com/en-ca/customer-billing-rates/bill-calculator.html (2023). Accessed: 3 December 2023.

[56] Enbridge Gas. Residential rates. https://www.enbridgegas.com/residential/my-account/rates (2024). Accessed: 20 March 2024.

[57] FortisBC. Residential natural gas rates. https://www.fortisbc.com/accounts-billing/billing-rates/natural-gas-rates/residential-rates (2024). Accessed: 20 March 2024.

[58] Government of Alberta. Regulated rates. https://ucahelps.alberta.ca/regulated-rates.aspx (2024). Accessed: 20 March 2024.

[59] Eastward Energy. Residential rates. https://eastwardenergy.com/for-home/rates/ (2024). Accessed: 20 March 2024.

[60] Manitoba Hydro. Billing rates. https://www.hydro.mb.ca/account/billing/rates/ (2024). Accessed: 20 March 2024.

[61] SaskEnergy. Residential rates. https://www.saskenergy.com/manage-account/rates/residential-rates (2024). Accessed: 20 March 2024.

[62] Énergir. Residential rates. https://energir.com/en/residential (2024). Accessed: 20 March 2024.

[63] ECCC. National Inventory Report 1990–2021: Greenhouse Gas Sources and Sinks in Canada. Tech. Rep., Environment and Climate Change Canada, Gatineau (2023).

[64] CER. Canada’s energy future 2023. Tech. Rep., Canada Energy Regulator, Calgary (2023).

[65] ECCC. Social cost of greenhouse gases: Estimates for carbon dioxide, methane, and nitrous oxide. Tech. Rep., Environment and Climate Change Canada, Gatineau (2023).

[66] Mills, E. Insurance in a climate of change. *Science* **309**, 1040–1044. 10.1126/science.1112121 (2005). 16099975 10.1126/science.1112121

[67] Rennert, K. et al. Comprehensive evidence implies a higher social cost of co2. *Nature* **610**, 687–692. 10.1038/s41586-022-05224-9 (2022). 36049503 10.1038/s41586-022-05224-9 PMC9605864

[68] Safdari, M., Dennis, K., Gharabaghi, B., Siddiqui, K. & Aliabadi, A. A. Implications of latent and sensible building energy loads using natural ventilation. *J. Build. Eng.* **96**, 110447. 10.1016/j.jobe.2024.110447 (2024).

[69] Marler, R. T. & Arora, J. S. The weighted sum method for multi-objective optimization: New insights. *Struct. Multidisc. Optim.* **41**, 853–862 (2010).

[70] ASHRAE. Standard 62.1: Ventilation and Acceptable Indoor Air Quality. Tech. Rep., American Society for Heating Refrigeration and Airconditioning Engineers, Peachtree Corners (2022).

[71] ASHRAE. Standard 90.2: Energy-Efficient Design of Low-Rise Residential Buildings. Tech. Rep., American Society for Heating Refrigeration and Airconditioning Engineers, Peachtree Corners (2018).

[72] NRCan. National Energy Code of Canada. Tech. Rep. (Office of Energy Efficiency, Natural Resources Canada, 2017).

[73] Smoucha, E., Fitzpatrick, K., Buckingham, S. & Knox, O. Life cycle analysis of the embodied carbon emissions from 14 wind turbines with rated powers between 50kw and 3.4mw. *J. Fundam. Renew. Energy Appl.* **6**, 10.4172/2090-4541.1000211 (2016).

[74] Finnegan, S., Jones, C. & Sharples, S. The embodied co2e of sustainable energy technologies used in buildings: A review article. *Energy Build.* **181**, 50–61. 10.1016/j.enbuild.2018.09.037 (2018).

[75] Hamot, L. et al. *Whole life carbon of photovoltaic installations*, Tech. Rep, Major Projects Association, Letchworth Garden City (2022).

[76] Electric, M. Tm65 embodied carbon calculation for puz-wz60vaa air source heat pump. Tech. Rep., Mitsubishi Electric Europe B.V., Hatfield (2023).

[77] Canada, S. Census profile, 2021 census of population. https://www12.statcan.gc.ca/census-recensement/2021/dp-pd/prof/index.cfm?Lang=E (2023). Accessed: 2025-08-02.

