# Supplementary material for: Building energy retrofits in Canada under government fiscal constraints
Source: Sci Rep. 2026 Apr 27;16:14663. doi: 10.1038/s41598-026-49147-1 (PMC13156324; doi:10.1038/s41598-026-49147-1)
Supplement: Supplementary file 1 — Supplementary Information. [file 41598_2026_49147_MOESM1_ESM.pdf]

Building Energy Retrofits in Canada Under Government Fiscal Constraints

Ali Madadizadeh[1], **Kamran Siddiqui**[2], **Amir A. Aliabadi**[1,*]

[1]Department of Mechanical Engineering, University of Guelph, Guelph, ON, Canada

[2]Department of Mechanical and Materials Engineering, Western University, London, Ontario, Canada

*Correspondence to aaliabad@uoguelph.ca

Supplementary Information

Economic and environmental analysis

Table S1. Variables used in economic evaluation[1]; note: some prices were obtained from local manufacturers, suppliers, and contractors.

Parameter	Units	Description	Value
i_n	%	Nominal interest rate	3.93
j	%	Inflation rate	2.17
P_{pv}	$\$m^{-2}$	Price of PV	384
P_{st}	$\$m^{-2}$	Price of ST	340
P_{bites}	$\$m^{-3}$	Price of BITES	200
P_{hp}	$\$m^{-2}$	Price of HP	38×2
P_{cr}	$\$m^{-2}$	Price of CR	8×2
P_{env}	$\$m^{-4}$ K^{-1} W	Price of increasing envelop thermal resistance	40
P_{inf}	\$	Price of reducing building infiltration	$1{,}500 \times 2$
C_{OMB}	$\$m^{-2}$	Base model operation and maintenance cost	1
OM_{pv}	$\$m^{-2}$	PV operation and maintenance cost	$0.01P_{pv}$
OM_{wt}	$\$m^{-2}$	WT operation and maintenance cost	$0.02P_{wt}$
OM_{st}	$\$m^{-2}$	ST operation and maintenance cost	$0.01P_{st}$
OM_{bites}	$\$m^{-3}$	BITES operation and maintenance cost	$0.01P_{bites}$
OM_{hp}	$\$m^{-2}$	HP operation and maintenance cost	$0.05P_{hp}$
OM_{cr}	$\$m^{-2}$	CR operation and maintenance cost	$0.1P_{cr}$
F_{SB}	-	Base model salvage factor	0.03
F_S	-	Retrofit model salvage factor	0.05
C_B	$\$m^{-2}$	Base model initial cost	5

Overall Objective Function

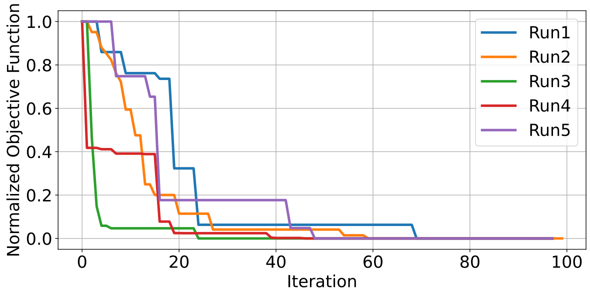

(a) 1E1F

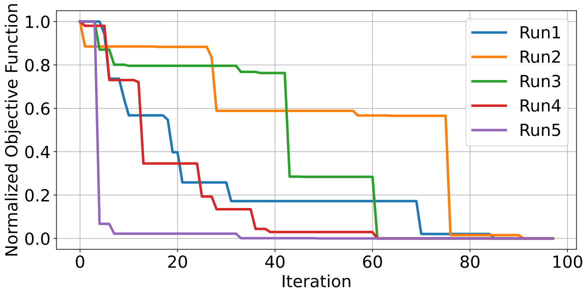

(b) 5E1F

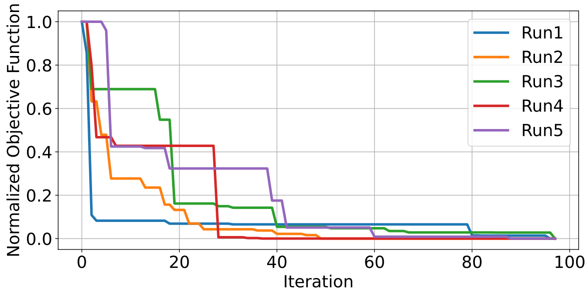

(c) 1E5F

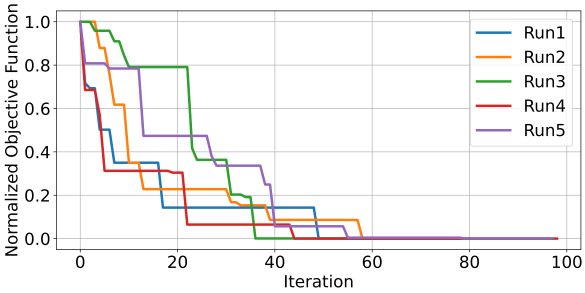

(d) 5E5F

Figure S1. Normalized overall objective function convergence trends for Toronto across different runs with varying energy price inflation rates.

Table S2. Sensitivity analysis scenarios for objective function weights (w_{GHG}, w_O, w_G).

Scenario (Weights)	GHG Savings [tonne CO_2e]	Owner Savings [$]	Government Savings [$]
S1 (0.333, 0.333, 0.333)	70	391	-1200
S2 (0.20, 0.60, 0.20)	68	1766	-2463
S3 (0.60, 0.20, 0.20)	73	1000	-2000
S4 (0.20, 0.20, 0.60)	68	600	-1200
S5 (0.45, 0.450, 0.10)	70	200	-1200
S6 (0.10, 0.45, 0.45)	75	400	-1500
S7 (0.45, 0.1, 0.45)	73	500	-1500

Retrofit Impact on Household Energy Burden

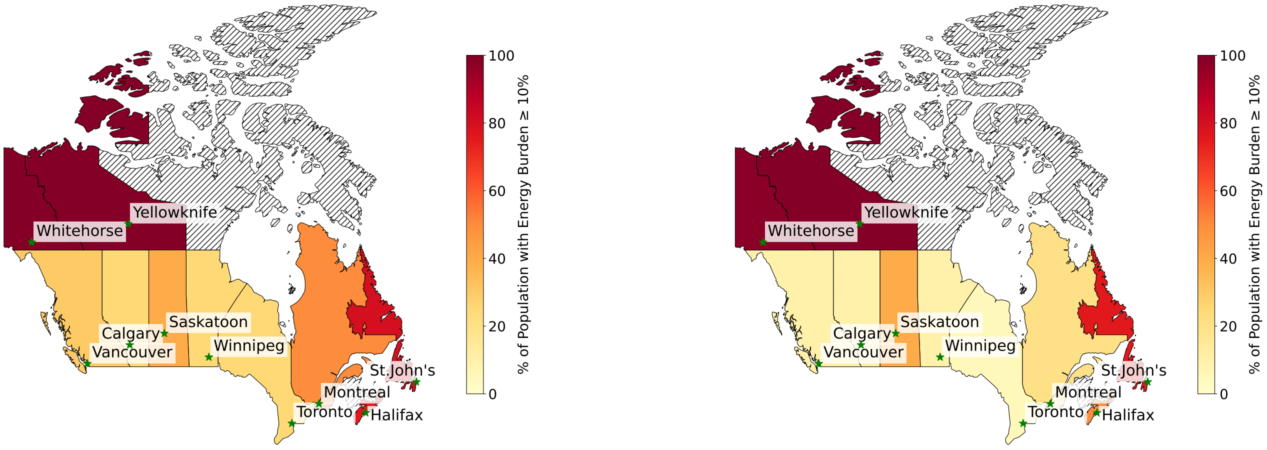

(a) Before retrofit. **(b)** After retrofit.

Figure S2. Energy burden comparison for 5E1F energy price inflation rates before and after retrofitting; map generated using python 3.10 and various libraries: geopandas 1.0.1, matplotlib 3.9.0, and unidecode 1.4.0 (https://www.python.org/).

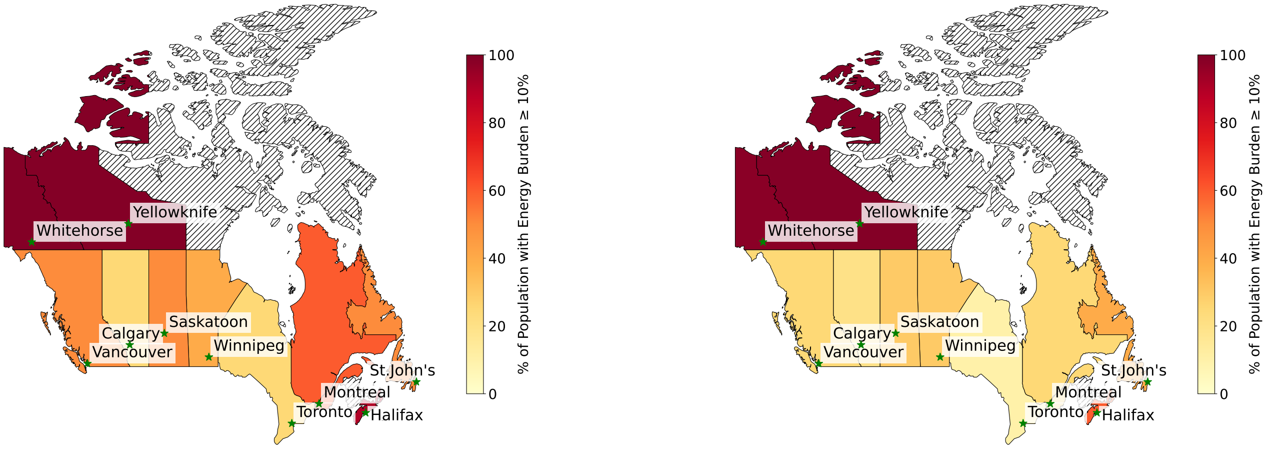

(a) Before retrofit. **(b)** After retrofit.

Figure S3. Energy burden comparison for 1E5F energy price inflation rates before and after retrofitting; map generated using python 3.10 and various libraries: geopandas 1.0.1, matplotlib 3.9.0, and unidecode 1.4.0 (https://www.python.org/).

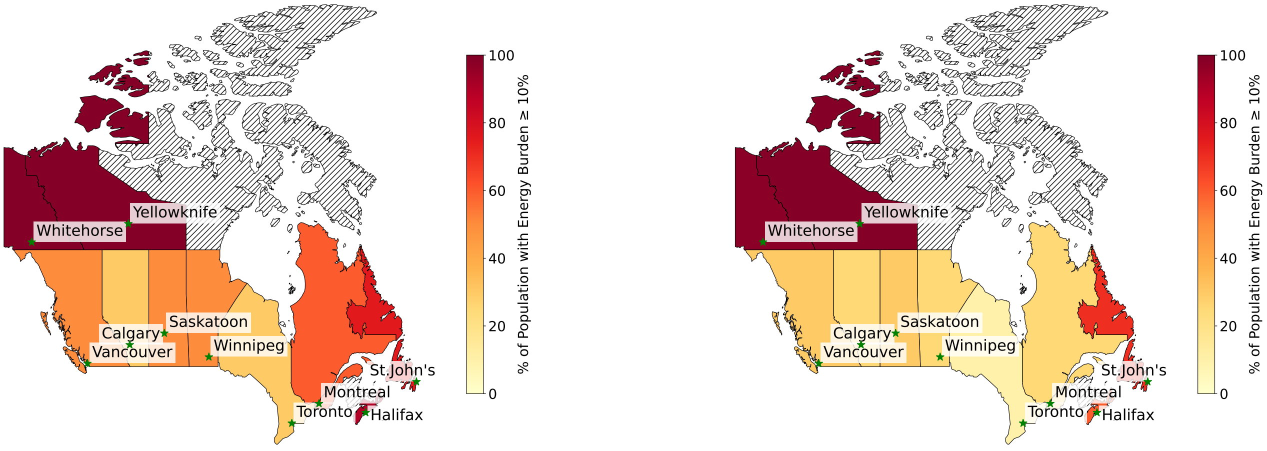

(a) Before retrofit.

(b) After retrofit.

Figure S4. Energy burden comparison for 5E5F energy price inflation rates before and after retrofitting; map generated using python 3.10 and various libraries: geopandas 1.0.1, matplotlib 3.9.0, and unidecode 1.4.0 (https://www.python.org/).

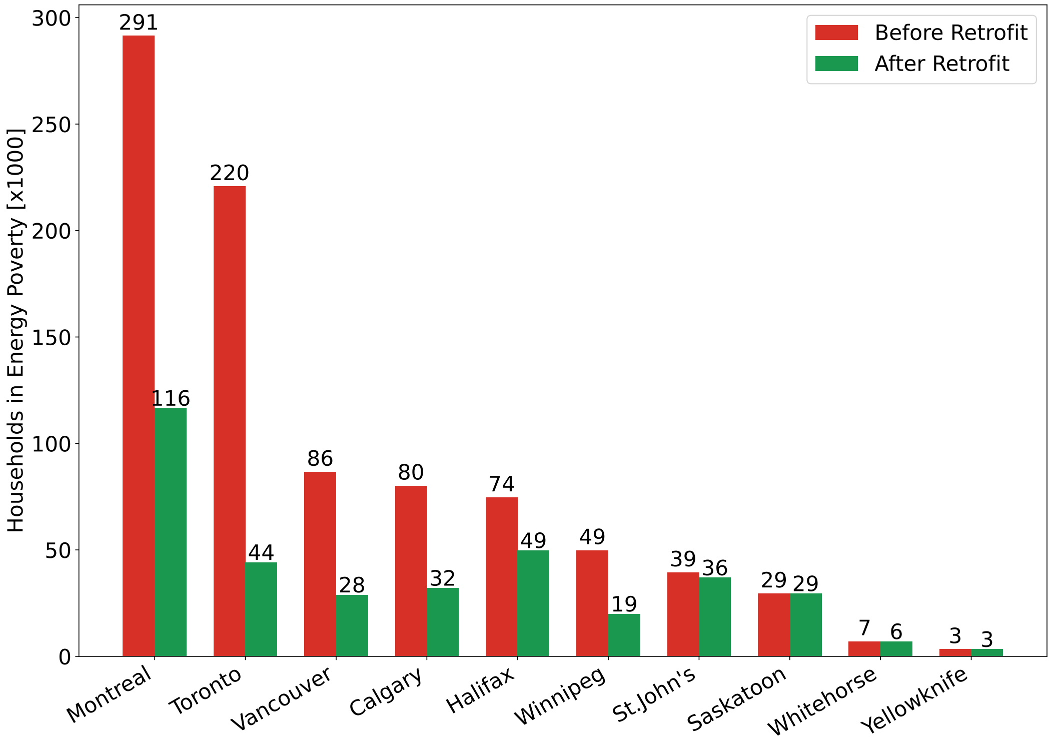

Figure S5. Number of households with energy poverty for 5E1F energy price inflation rates before and after retrofit.

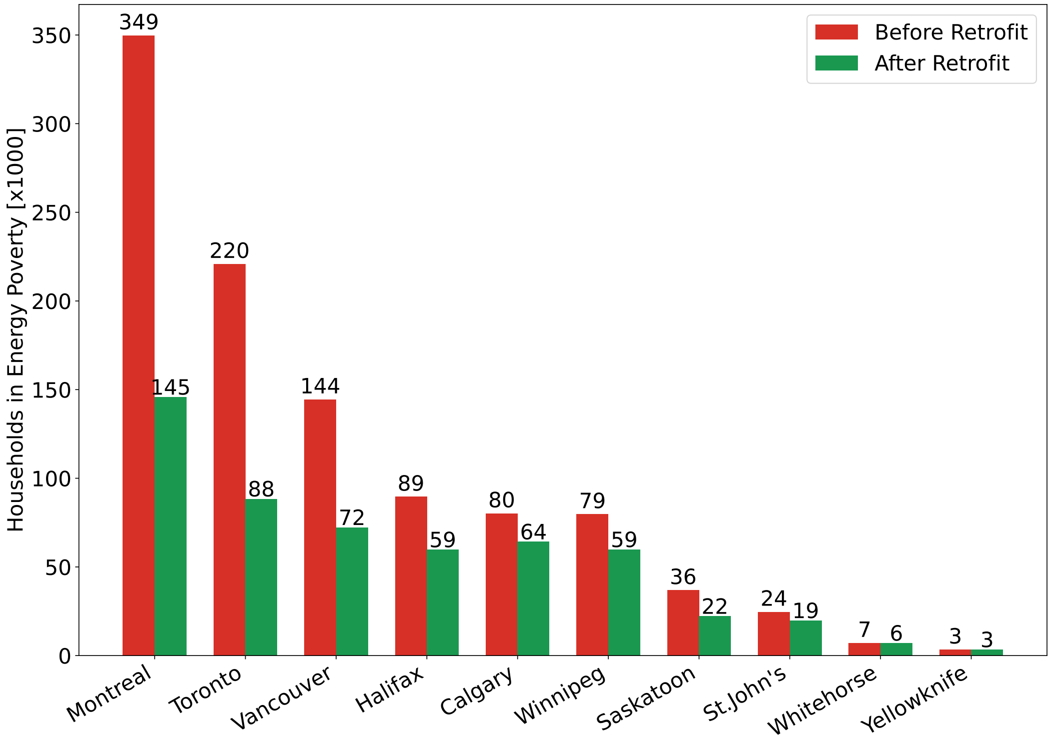

Figure S6. Number of households with energy poverty for 1E5F energy price inflation rates before and after retrofit.

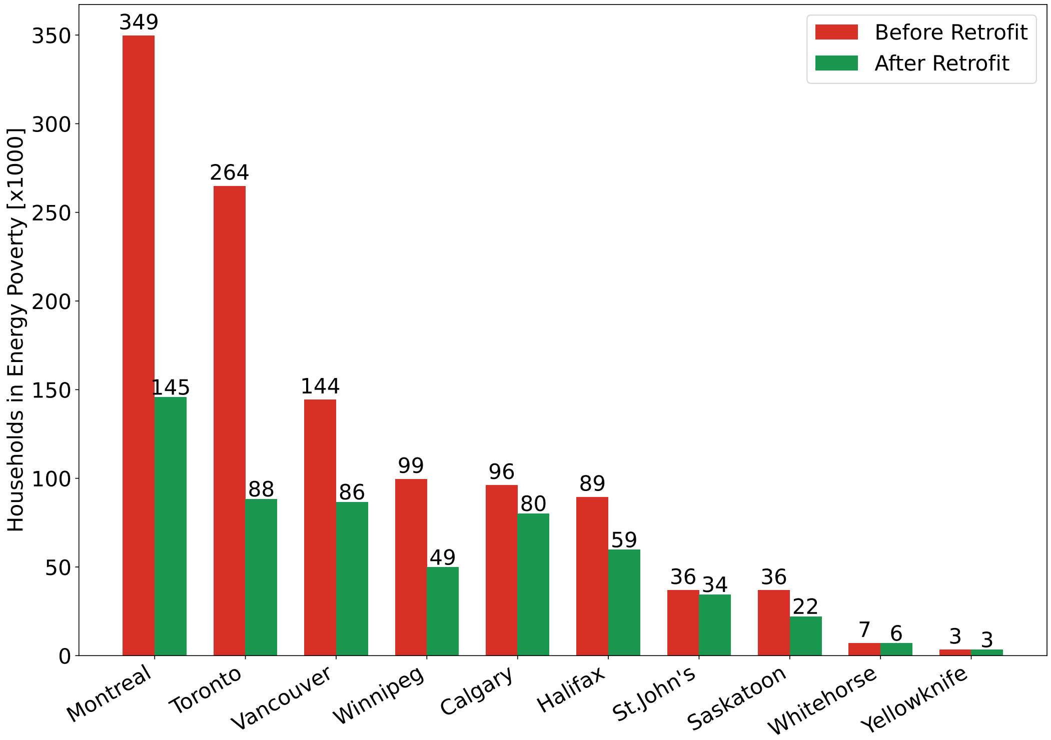

Figure S7. Number of households with energy poverty for 5E5F energy price inflation rates before and after retrofit.

Optimized economic parameters

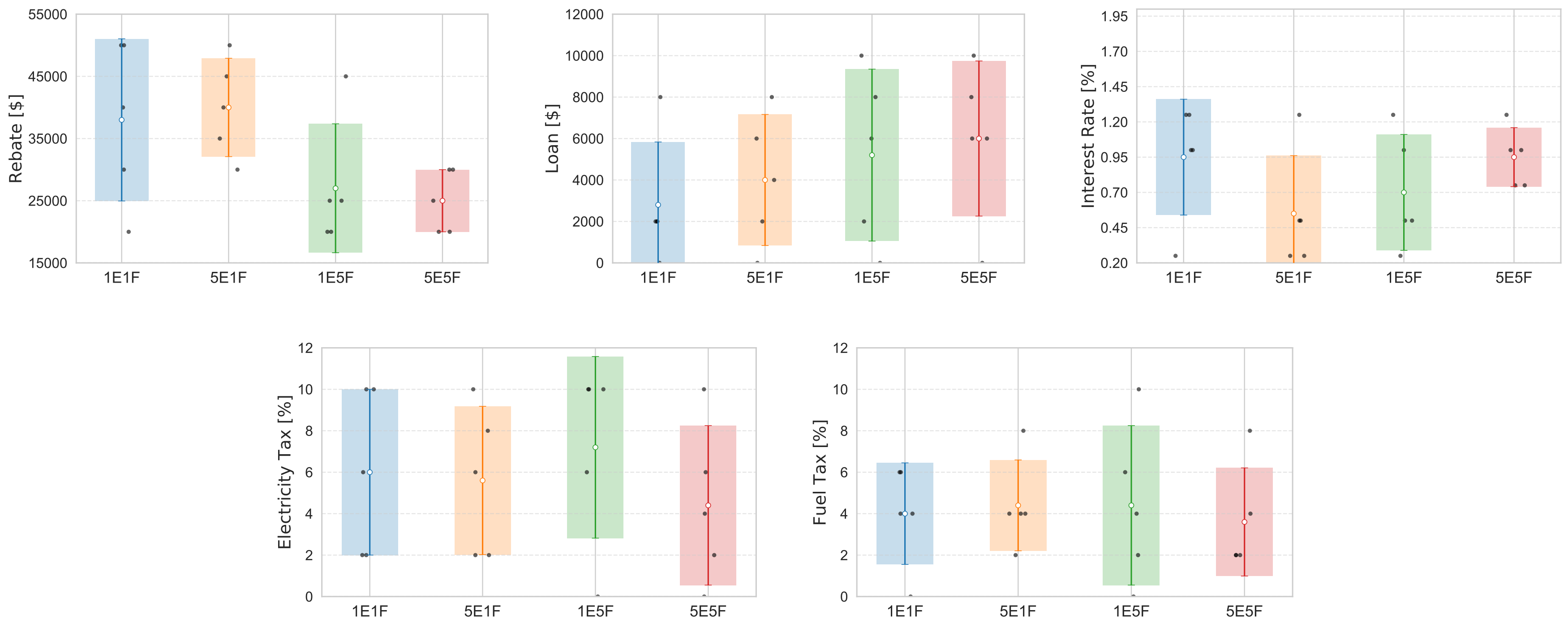

Figure S8. Optimized economic parameters for Vancouver across different energy price inflation scenarios.

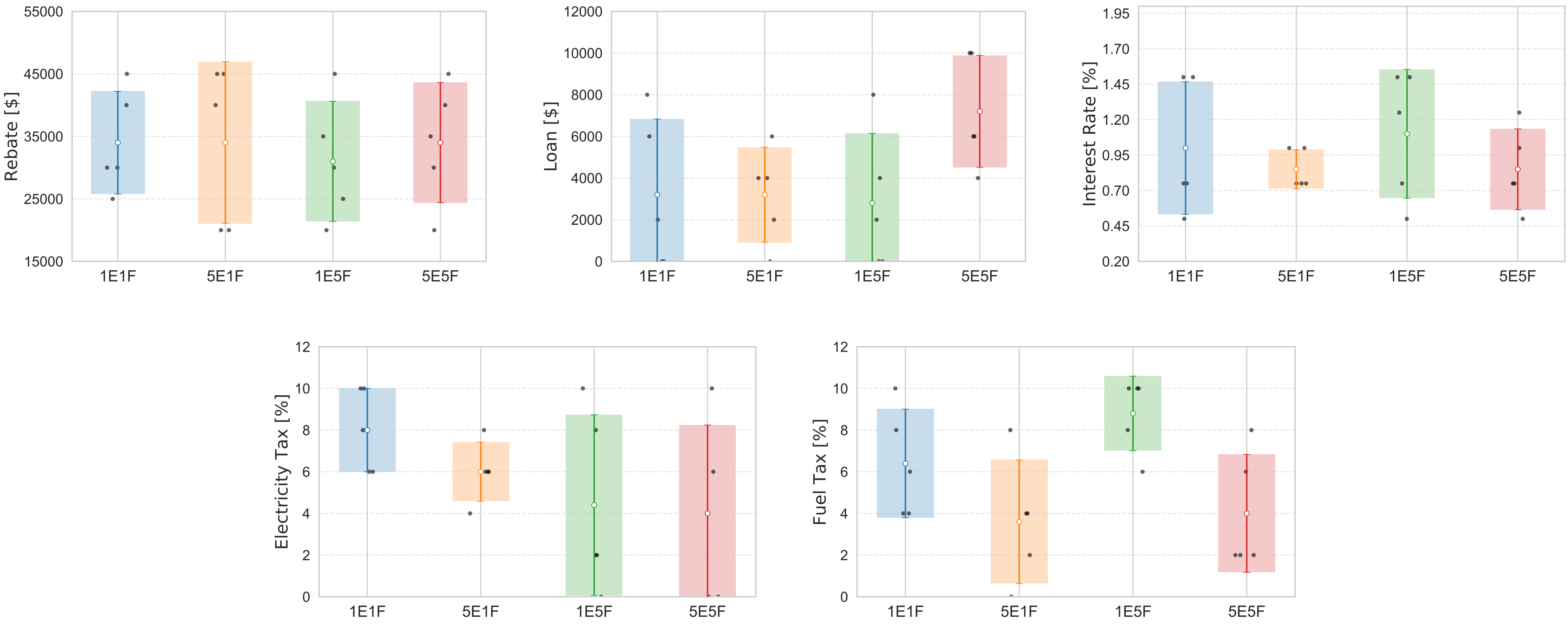

Figure S9. Optimized economic parameters for Toronto across different energy price inflation scenarios.

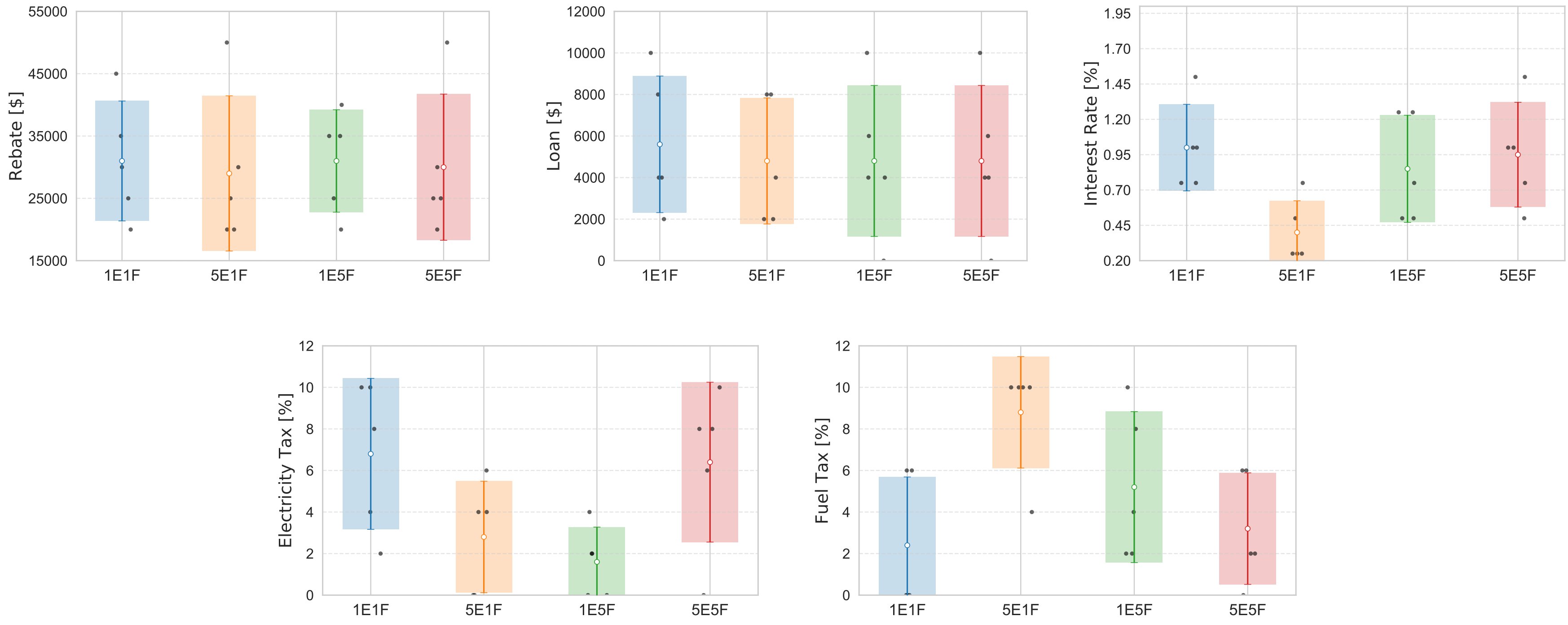

Figure S10. Optimized economic parameters for Halifax across different energy price inflation scenarios.

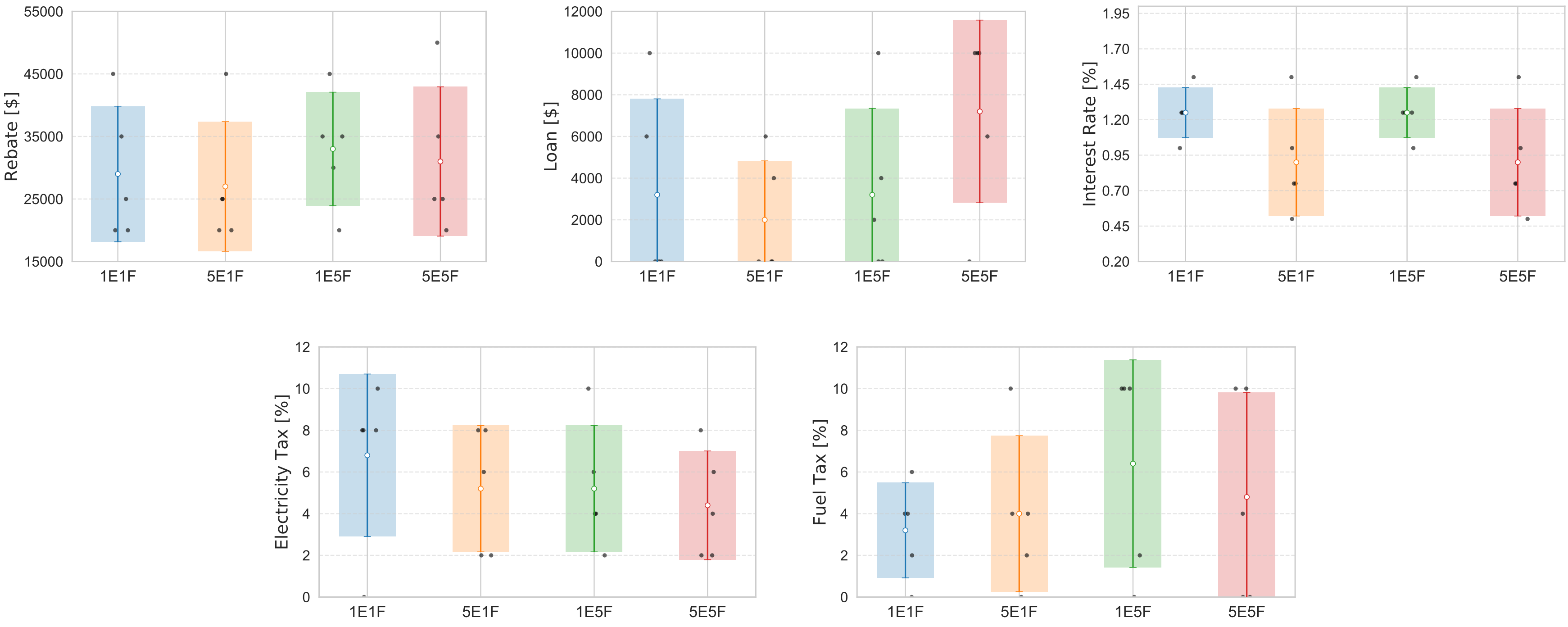

Figure S11. Optimized economic parameters for Montreal across different energy price inflation scenarios.

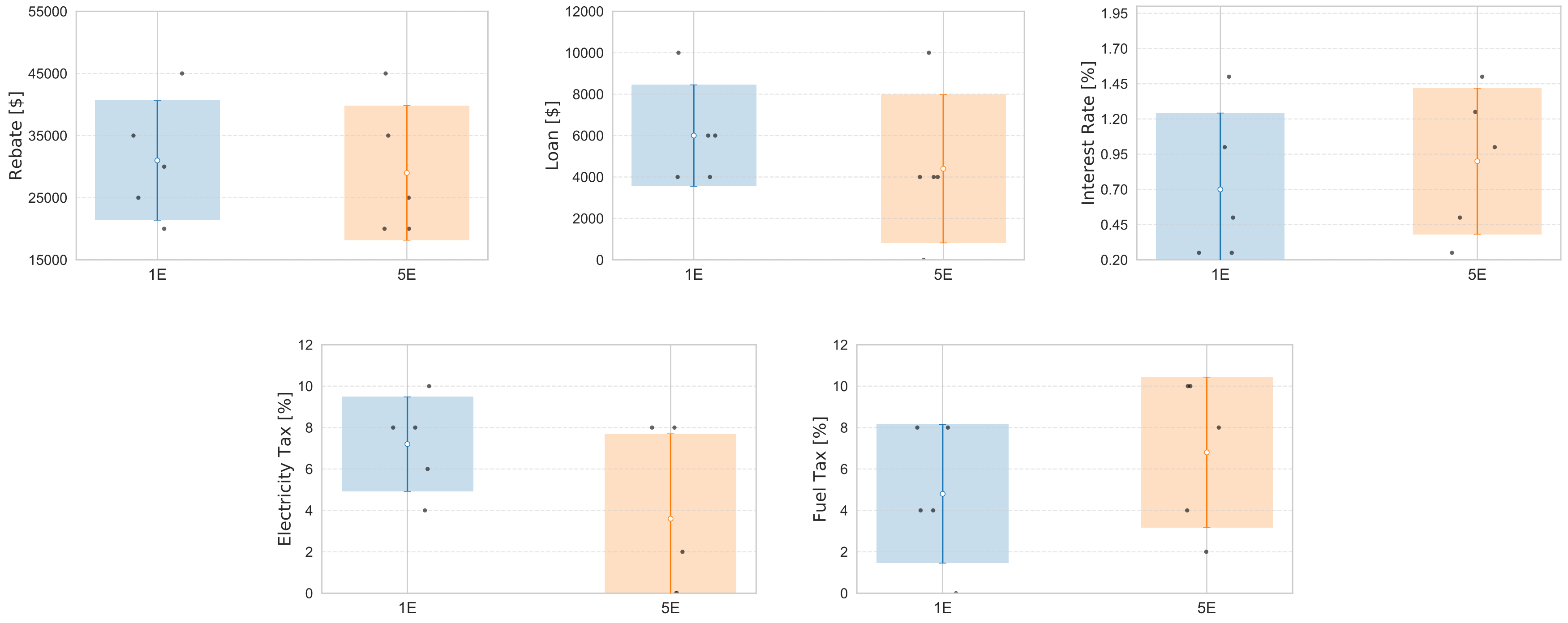

Figure S12. Optimized economic parameters for St. John's across different energy price inflation scenarios.

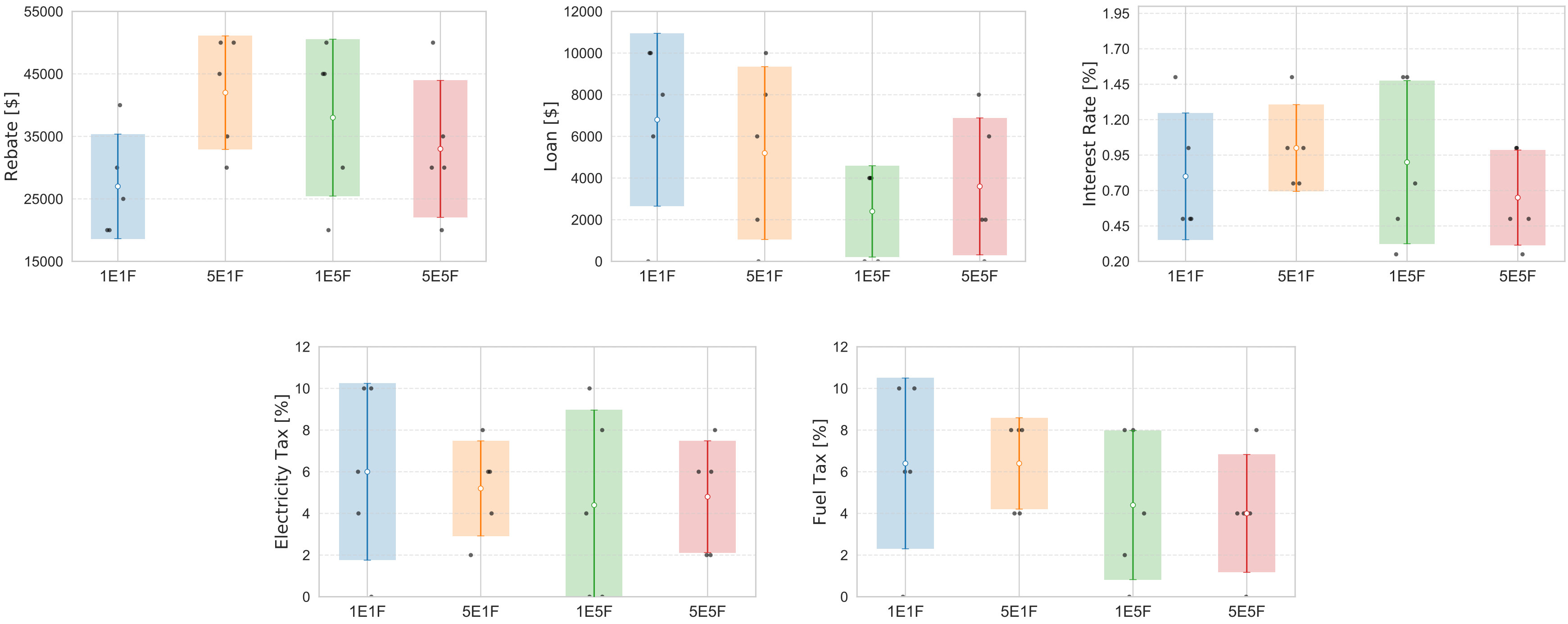

Figure S13. Optimized economic parameters for Calgary across different energy price inflation scenarios.

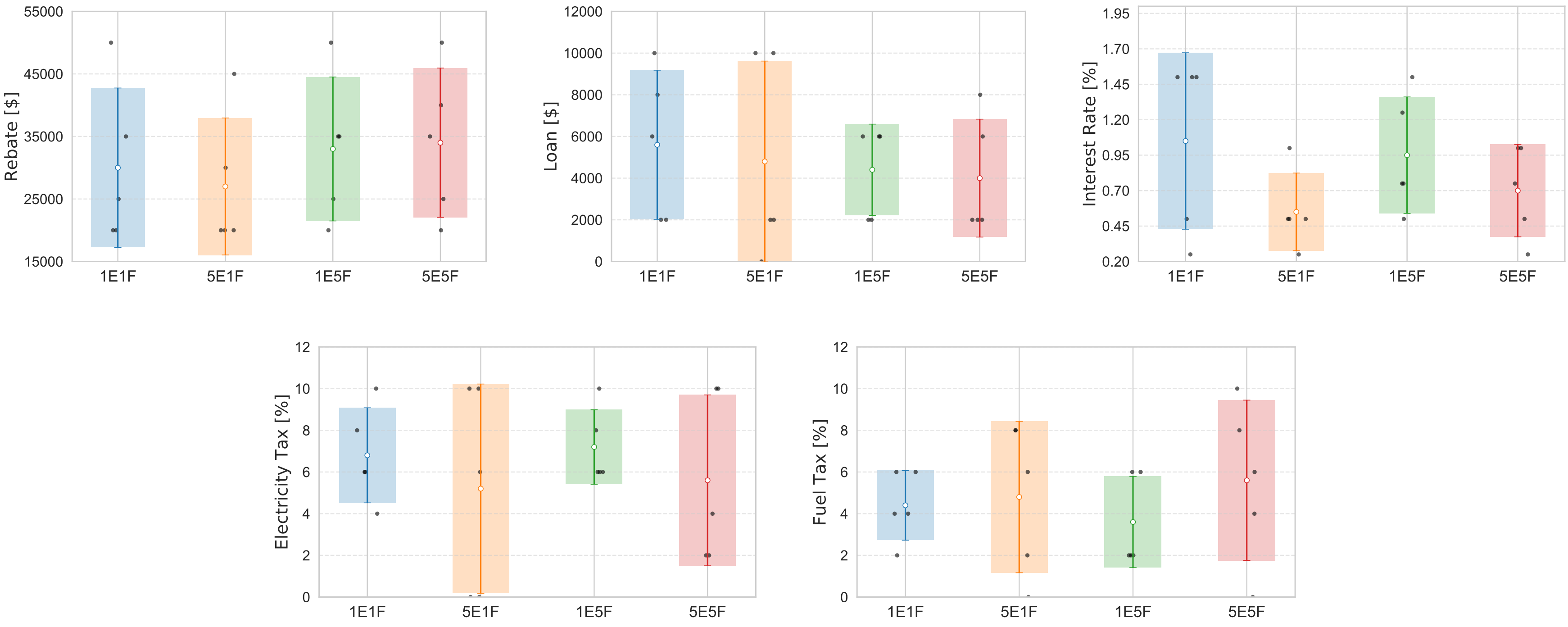

Figure S14. Optimized economic parameters for Saskatoon across different energy price inflation scenarios.

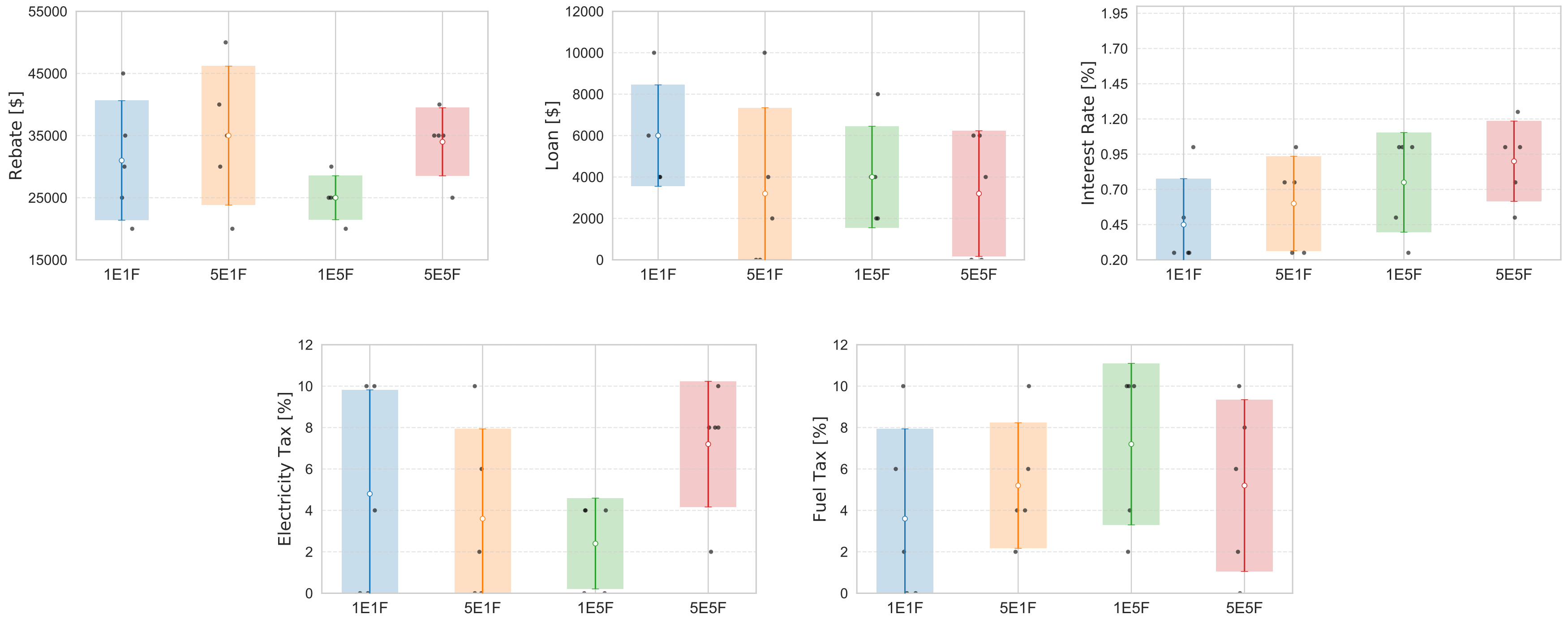

Figure S15. Optimized economic parameters for Winnipeg across different energy price inflation scenarios.

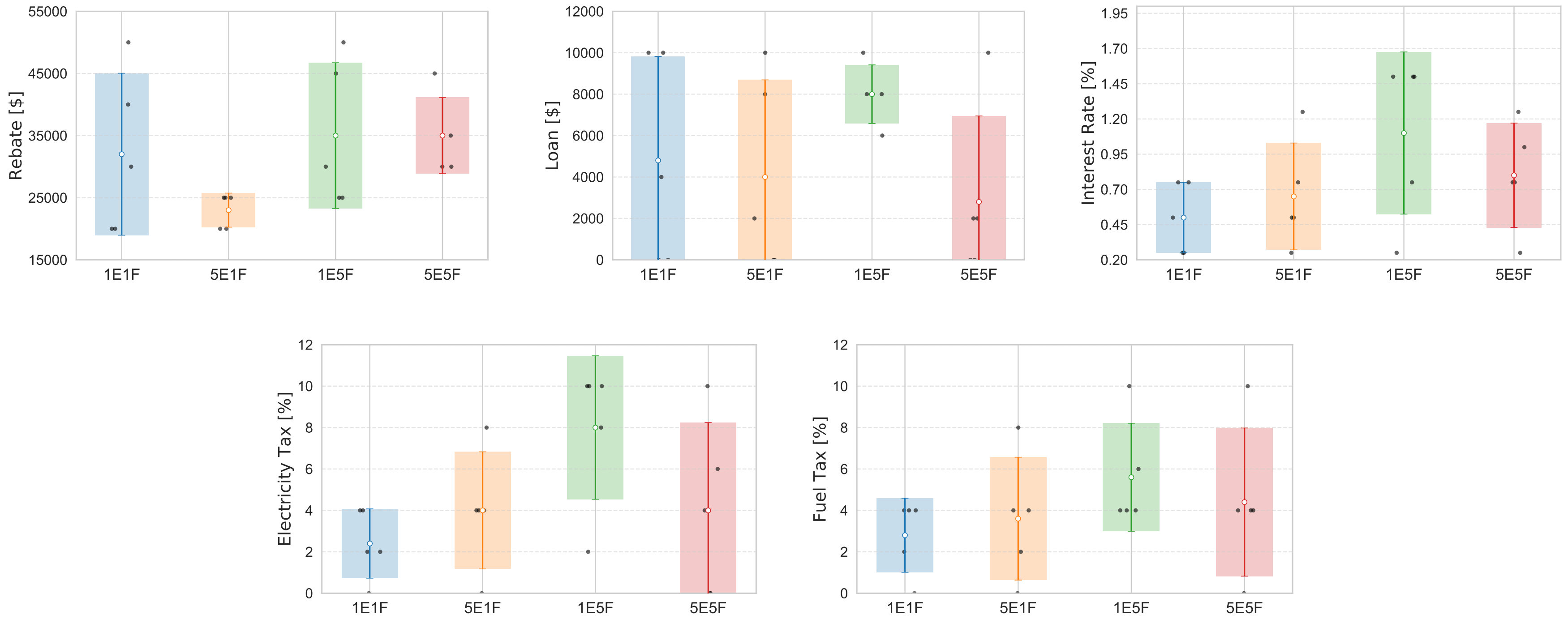

Figure S16. Optimized economic parameters for Whitehorse across different energy price inflation scenarios.

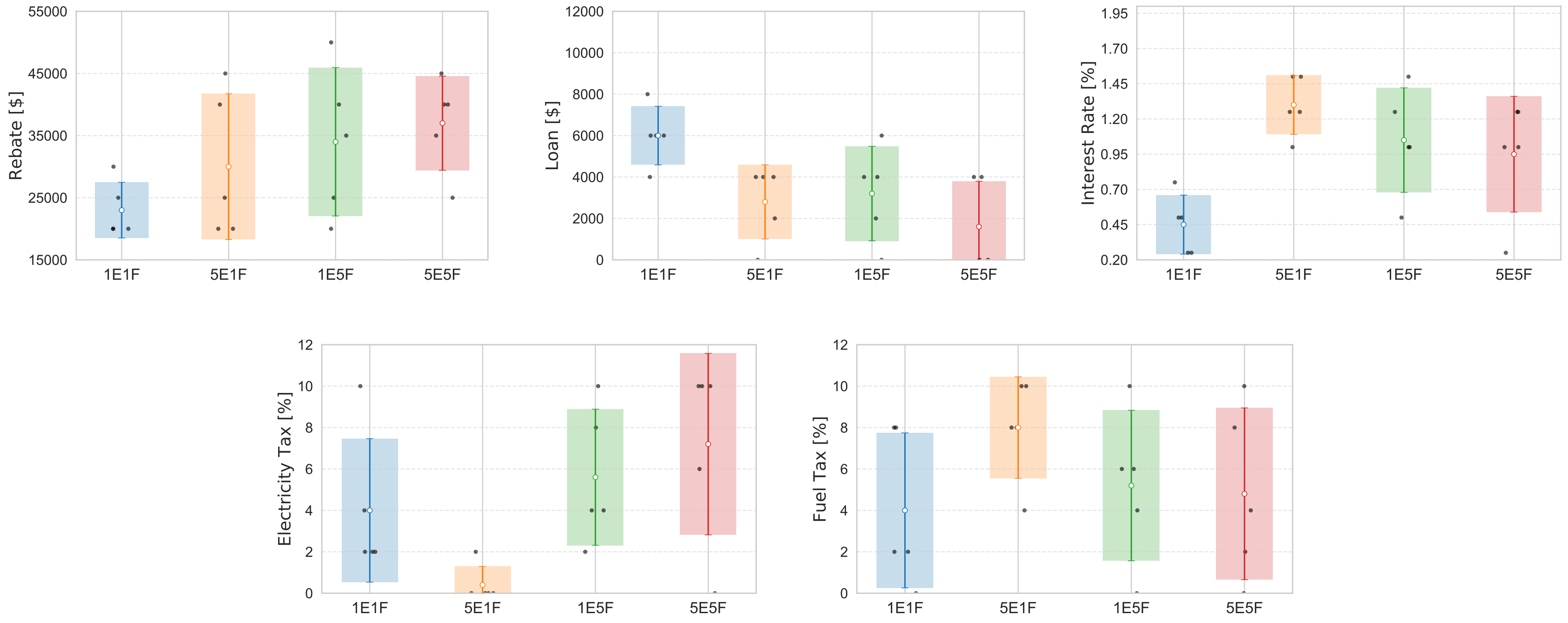

Figure S17. Optimized economic parameters for Yellowknife across different energy price inflation scenarios.

Optimized building parameters

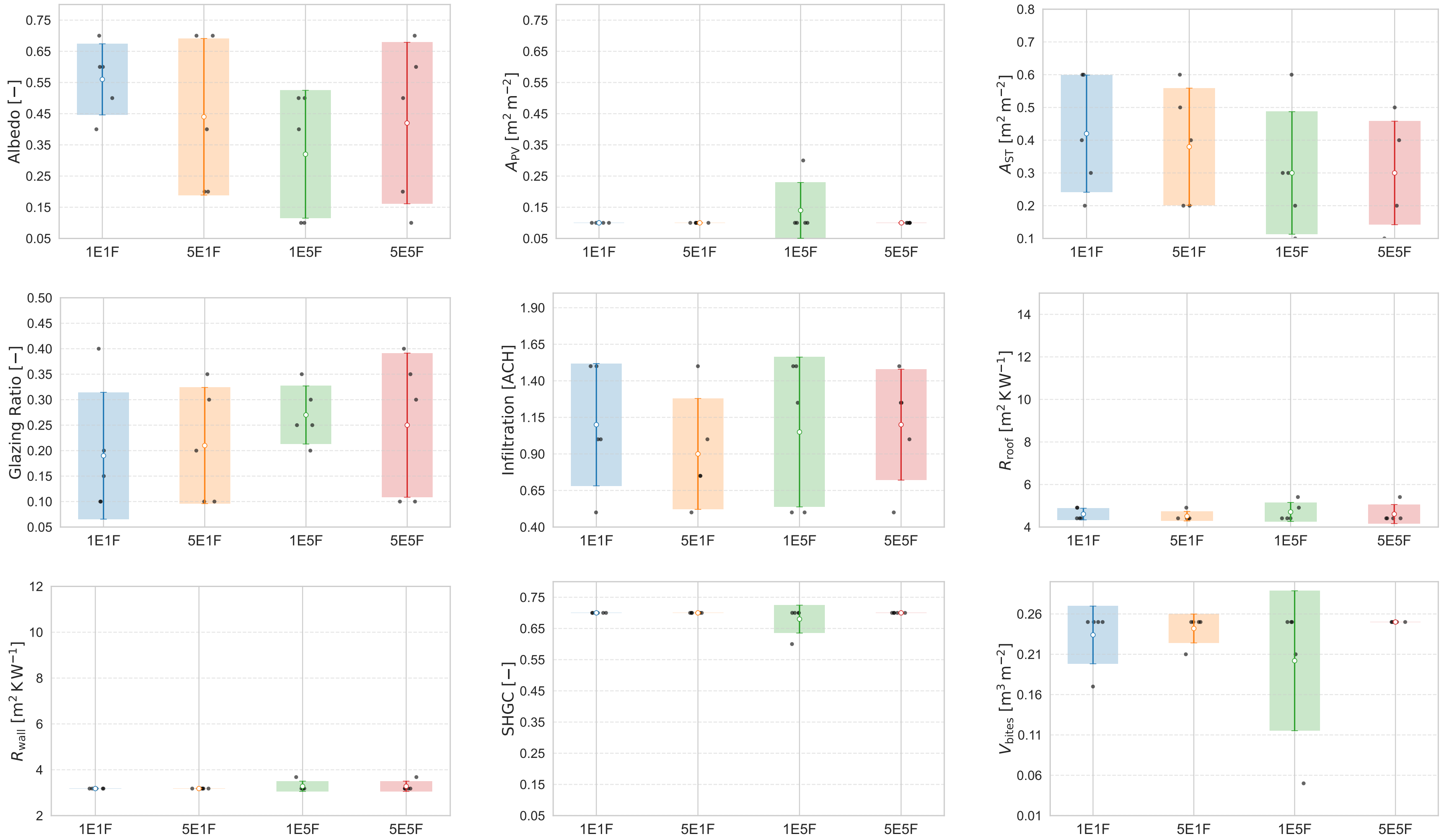

Figure S18. Optimized building parameters for Vancouver across different runs with varying energy price inflation rates.

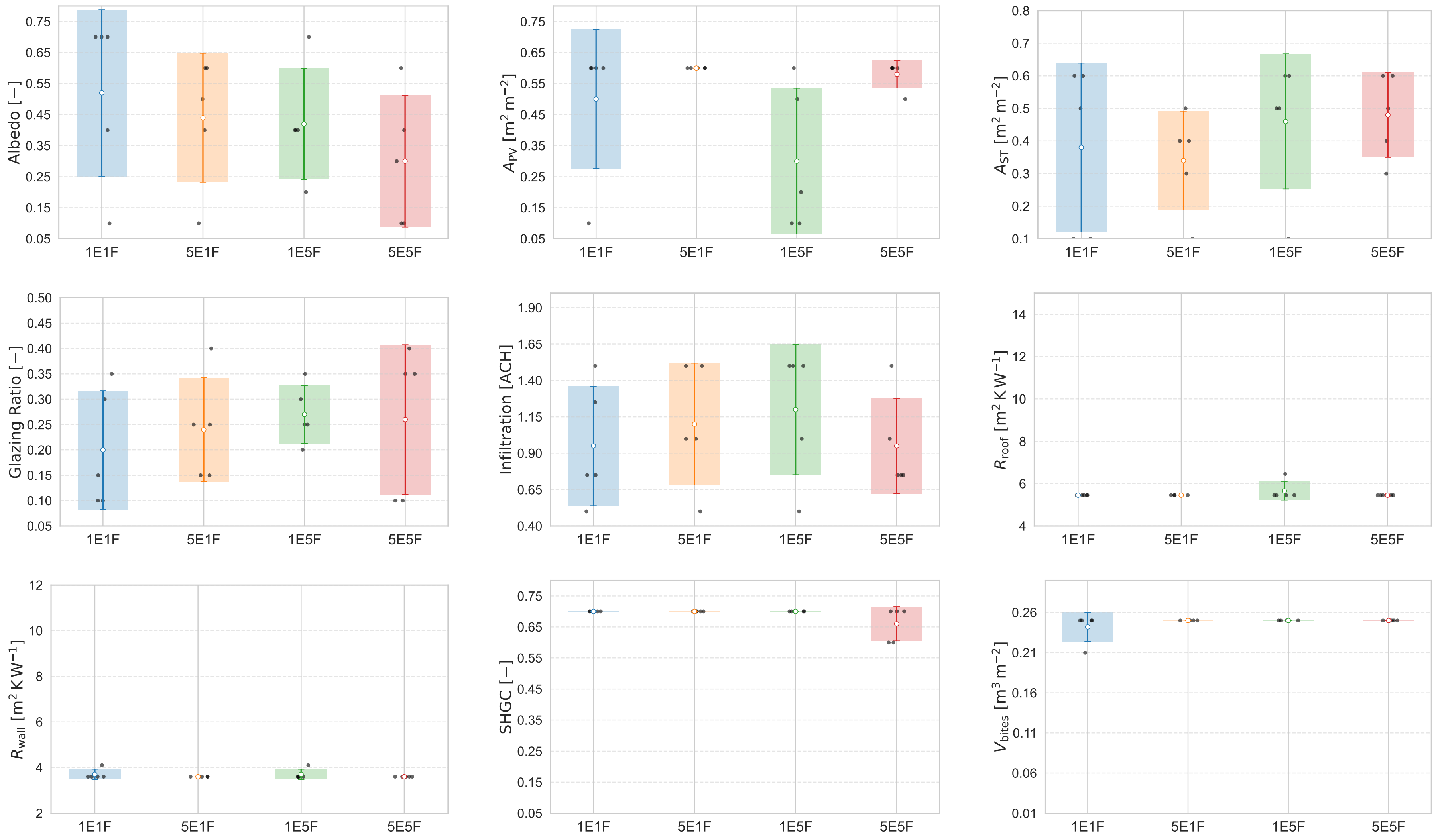

Figure S19. Optimized building parameters for Toronto across different runs with varying energy price inflation rates.

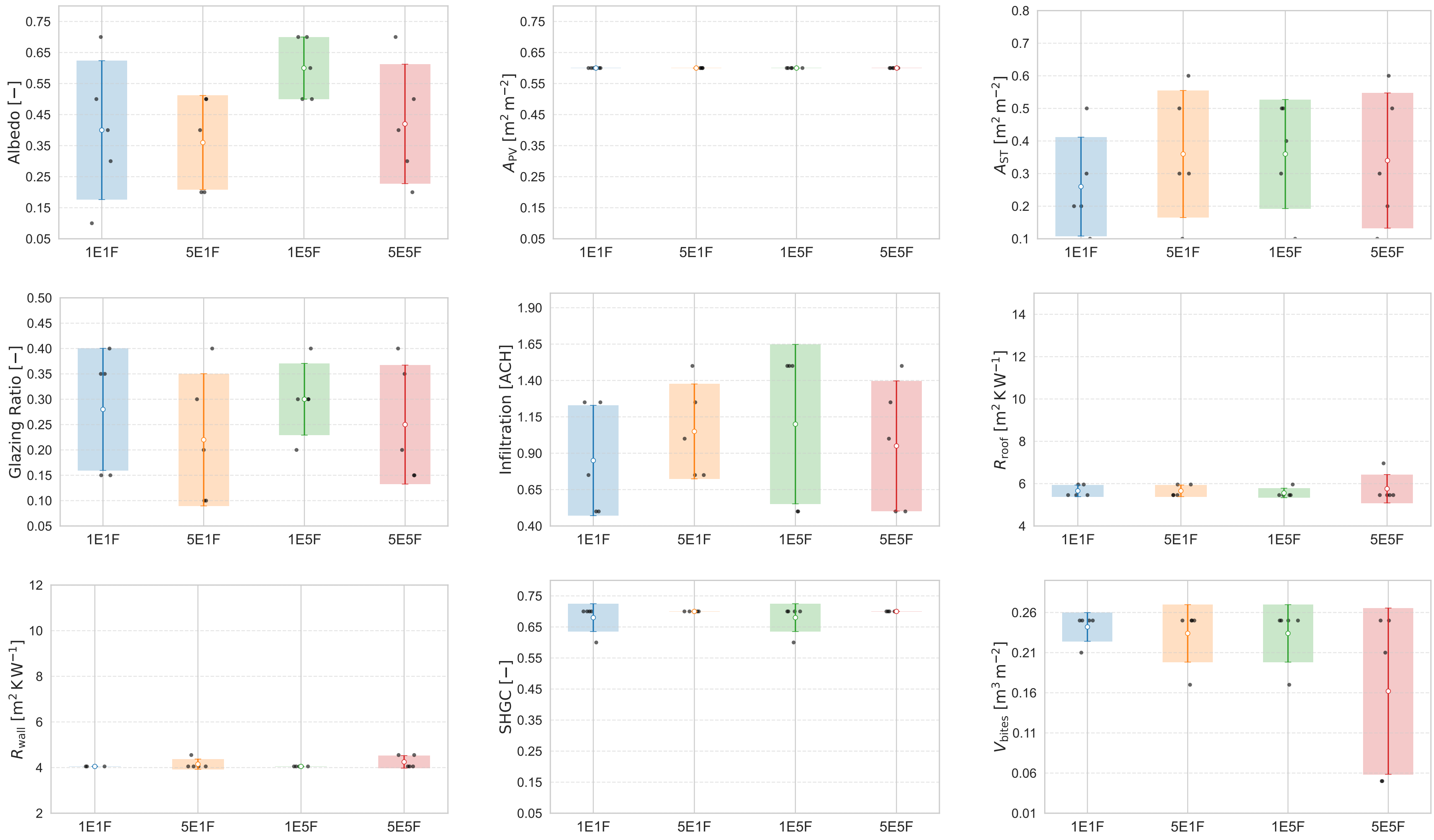

Figure S20. Optimized building parameters for Halifax across different runs with varying energy price inflation rates.

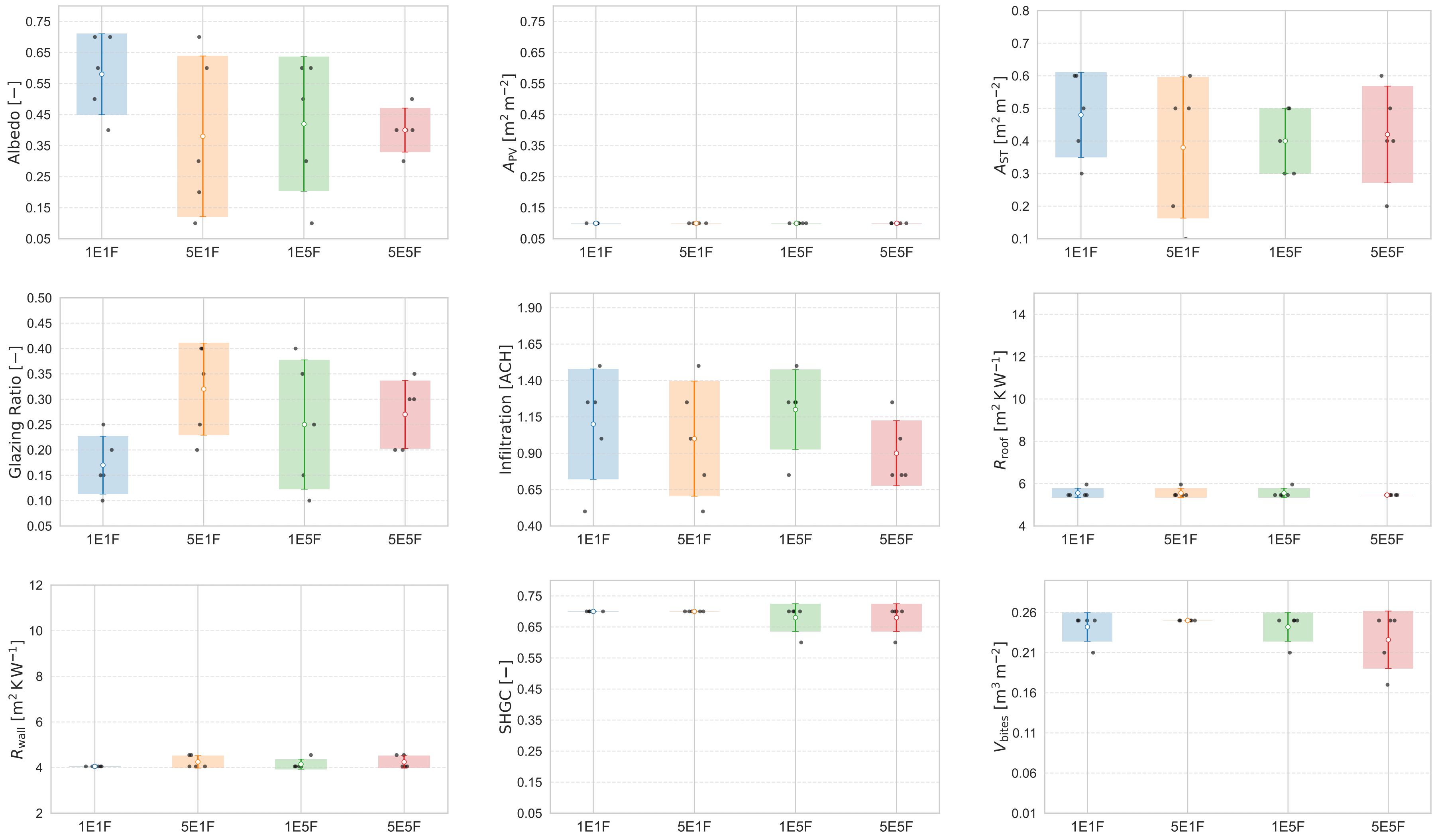

Figure S21. Optimized building parameters for Montreal across different runs with varying energy price inflation rates.

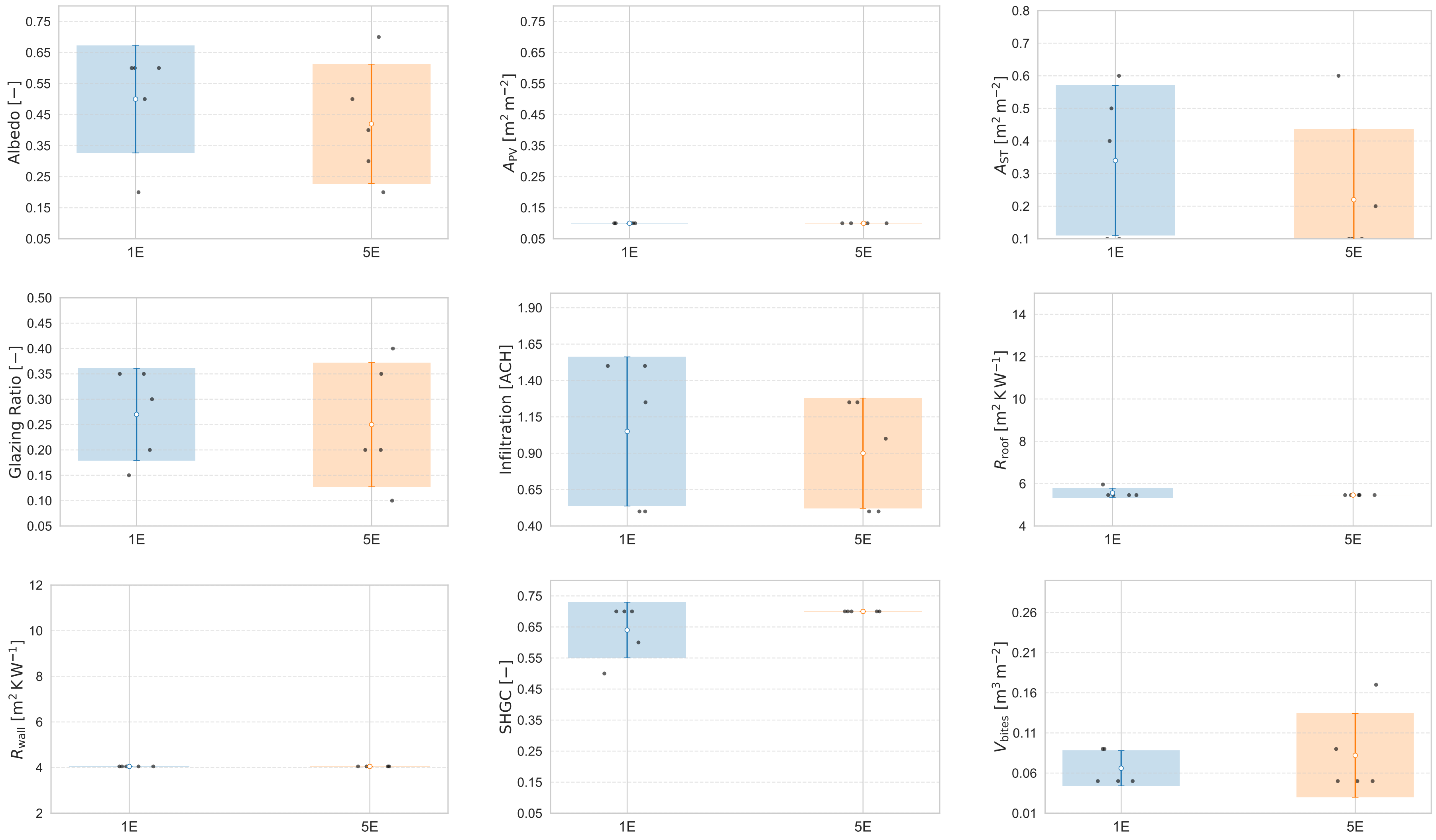

Figure S22. Optimized building parameters for St.John's across different runs with varying energy price inflation rates.

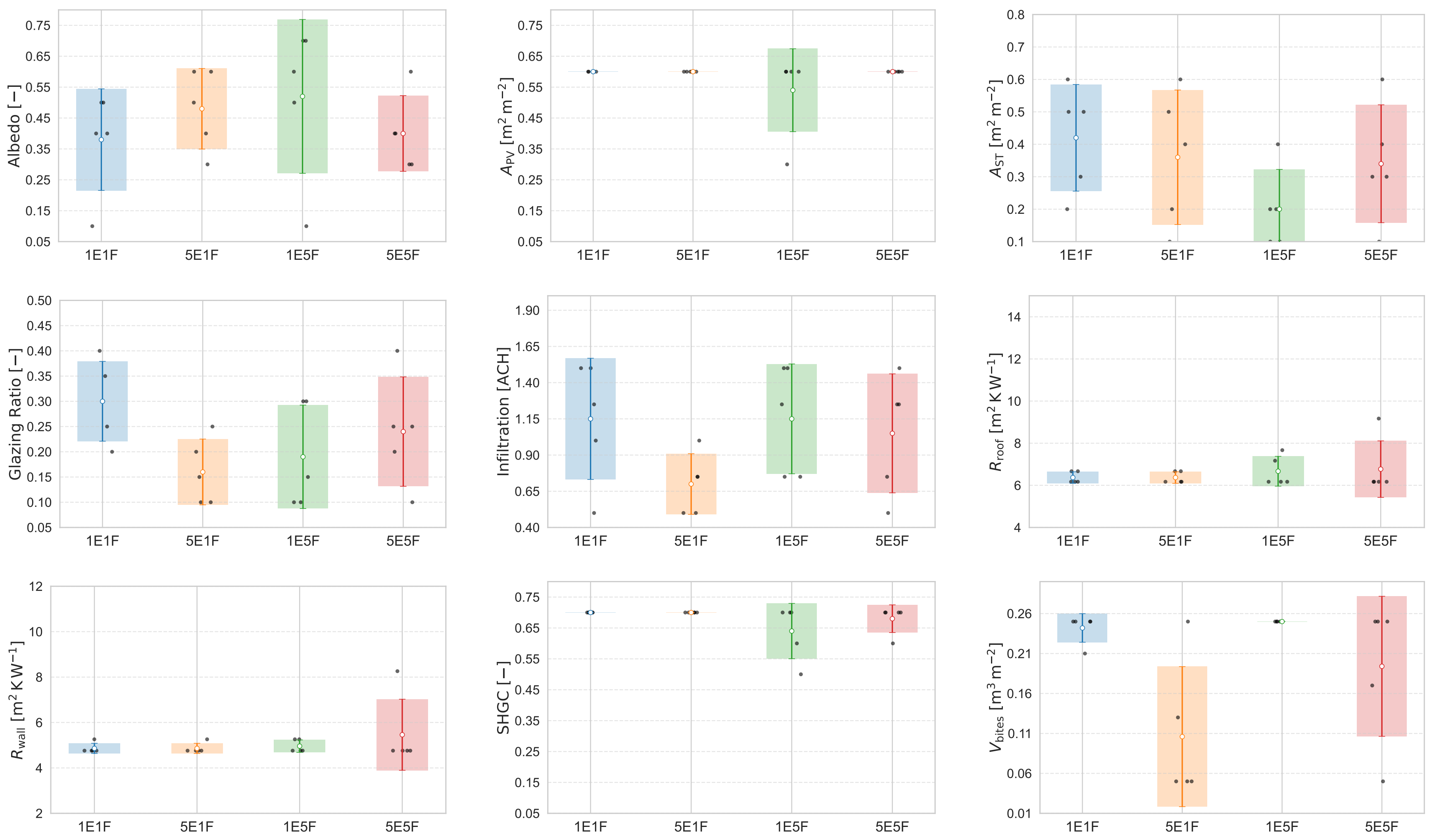

Figure S23. Optimized building parameters for Calgary across different runs with varying energy price inflation rates.

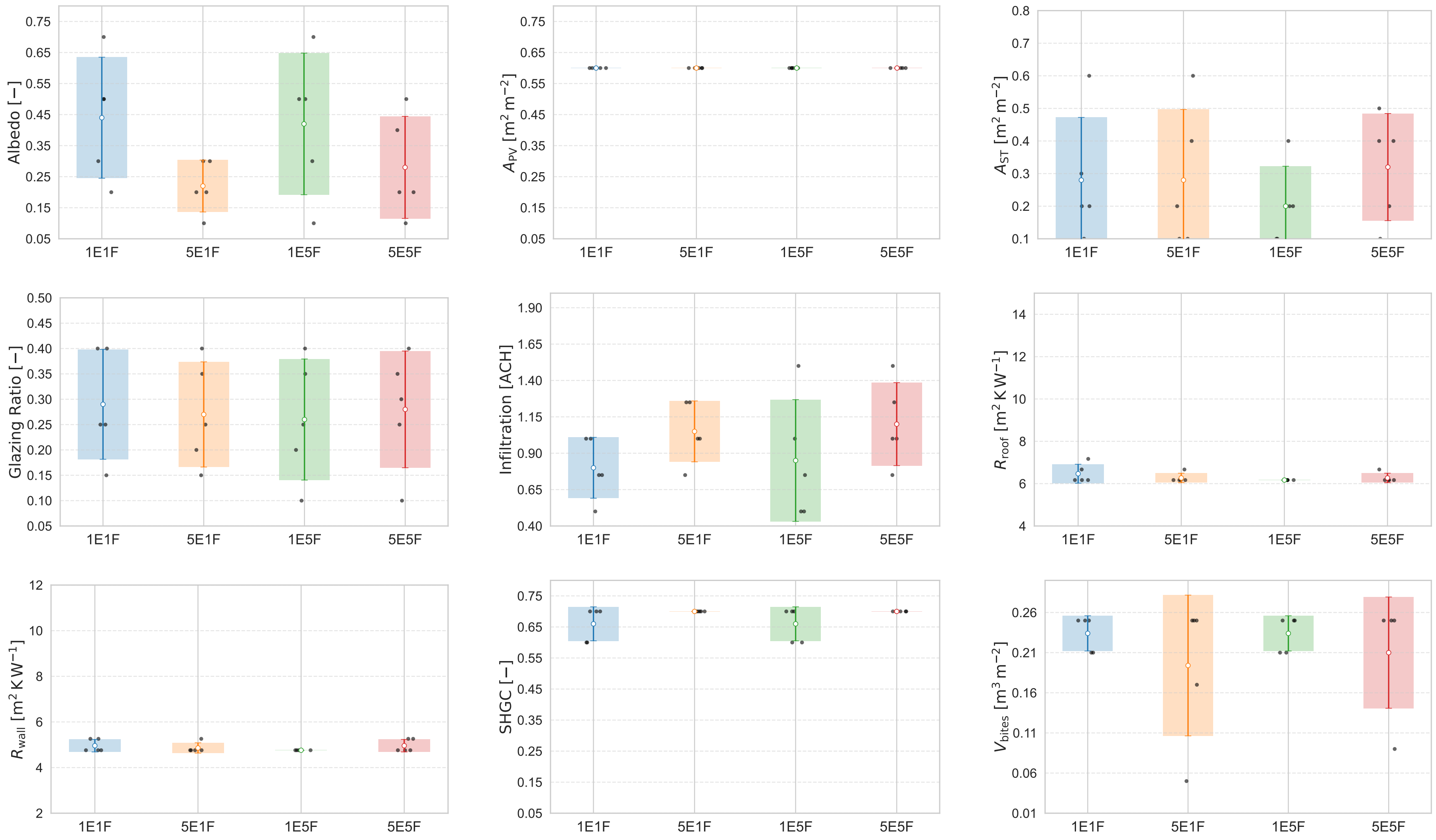

Figure S24. Optimized building parameters for Saskatoon across different runs with varying energy price inflation rates.

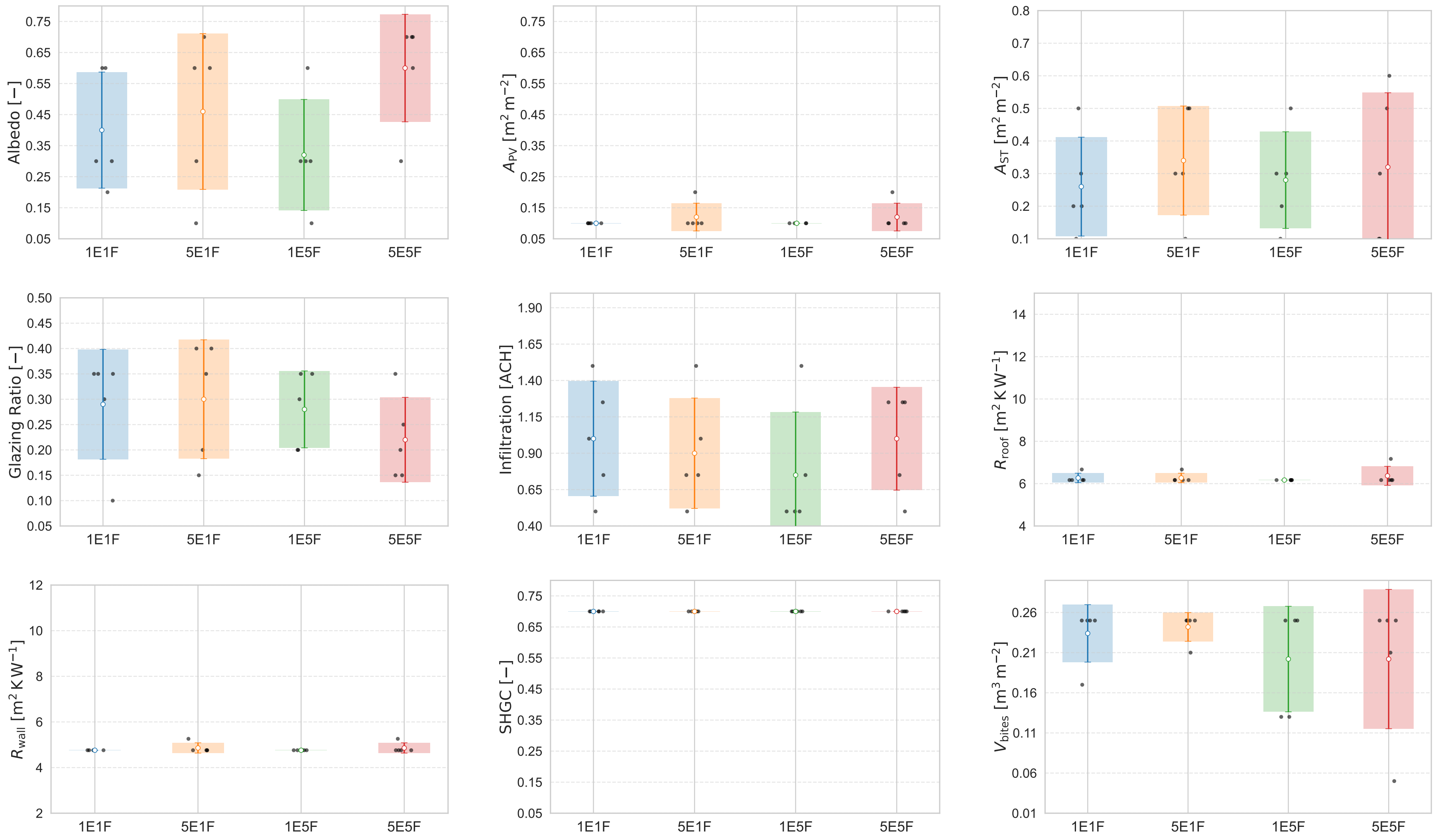

Figure S25. Optimized building parameters for Winnipeg across different runs with varying energy price inflation rates.

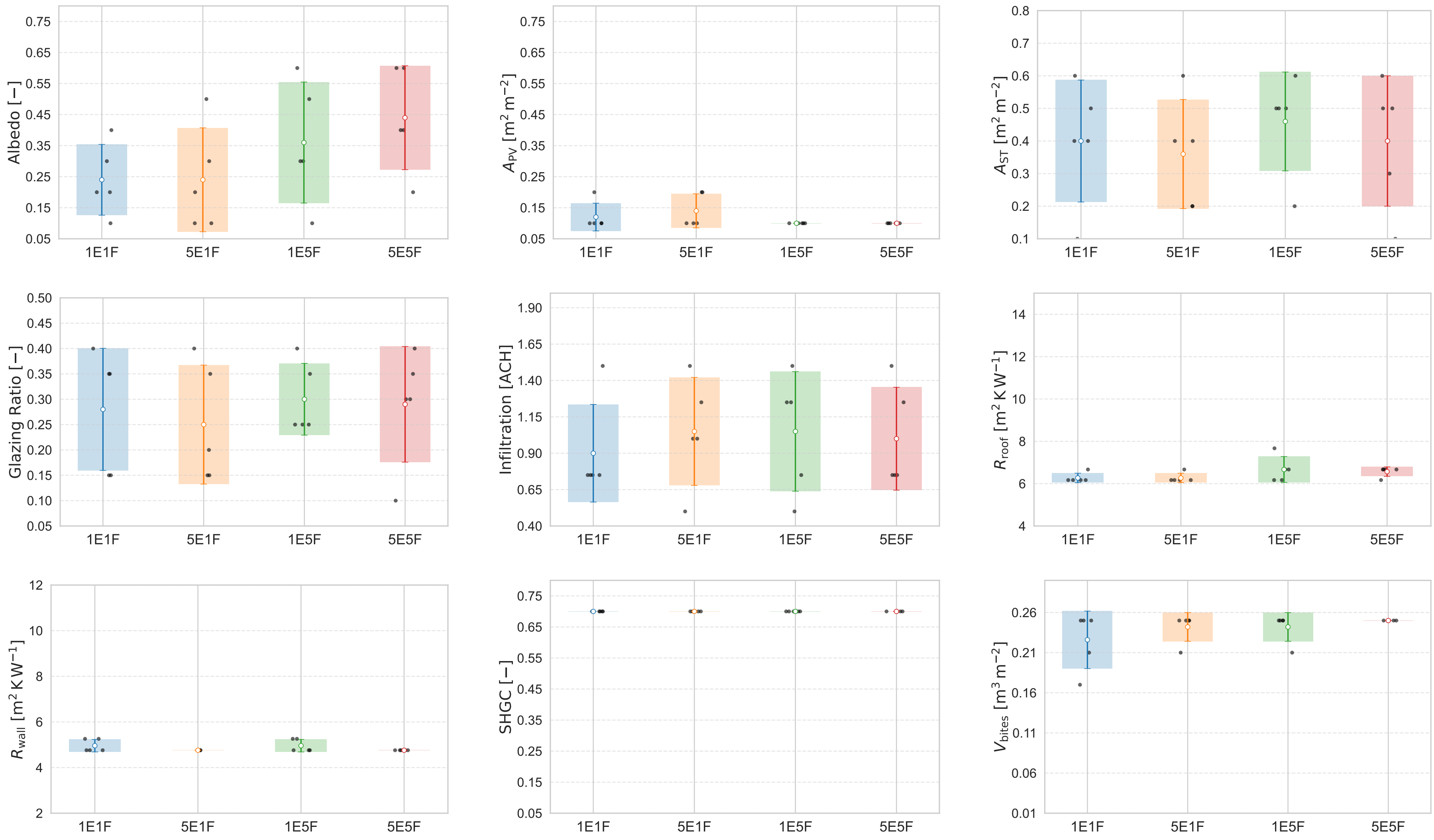

Figure S26. Optimized building parameters for Whitehorse across different runs with varying energy price inflation rates.

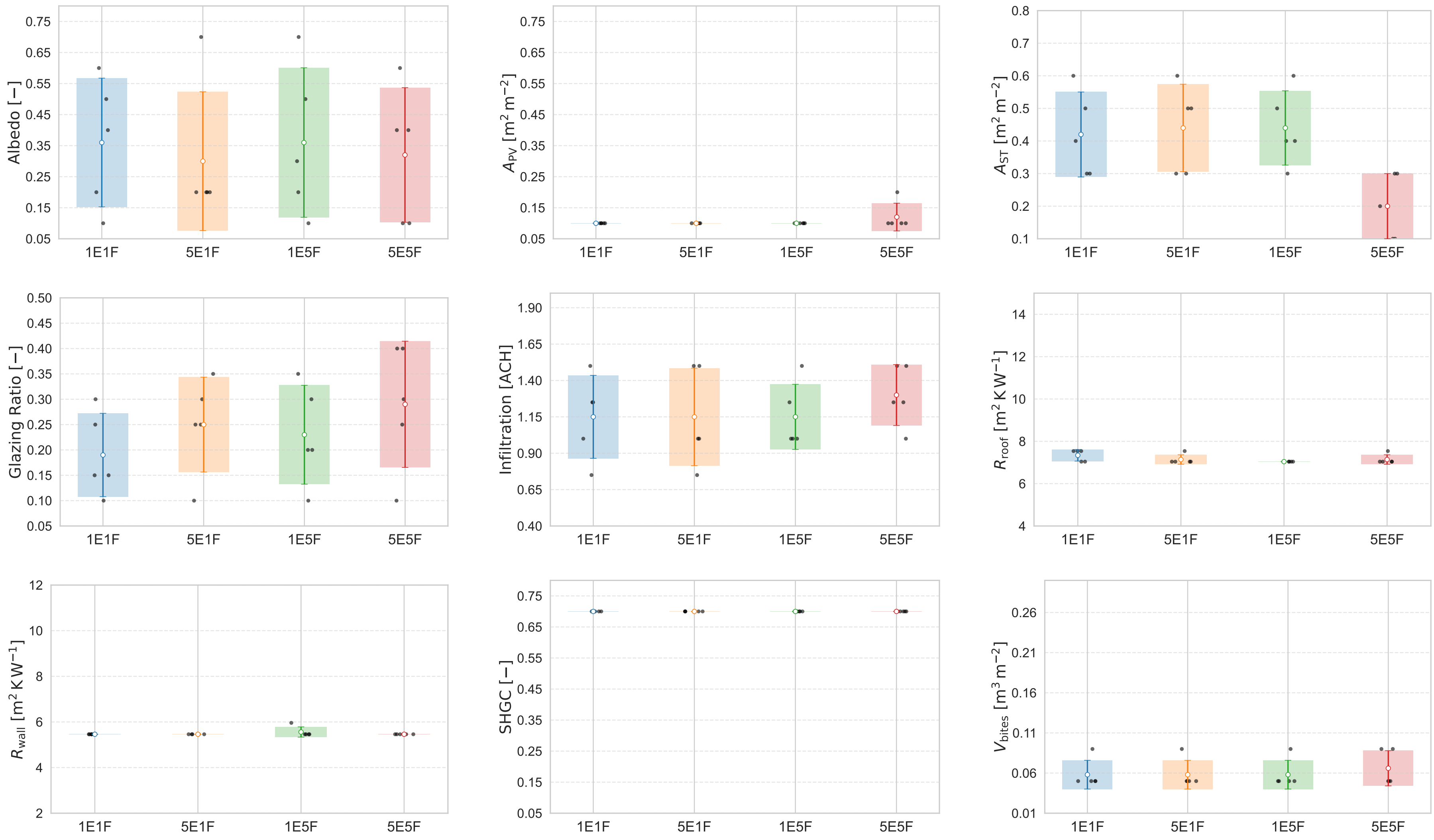

Figure S27. Optimized building parameters for Yellowknife across different runs with varying energy price inflation rates.

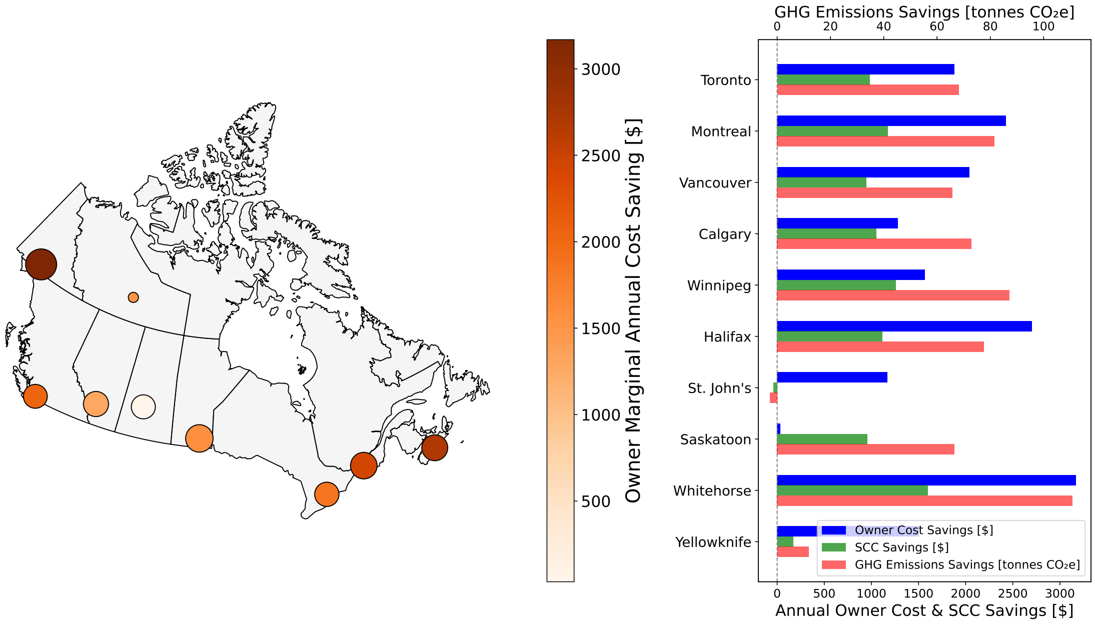

Figure S28. Total GHG and annual cost and SCC saving after building retrofit under 5E1F energy price inflation rates. The panel shows GHG emissions savings (size) and SCC savings (color); map generated using python 3.10 and various libraries: geopandas 1.0.1, matplotlib 3.9.0, and unidecode 1.4.0 (https://www.python.org/).

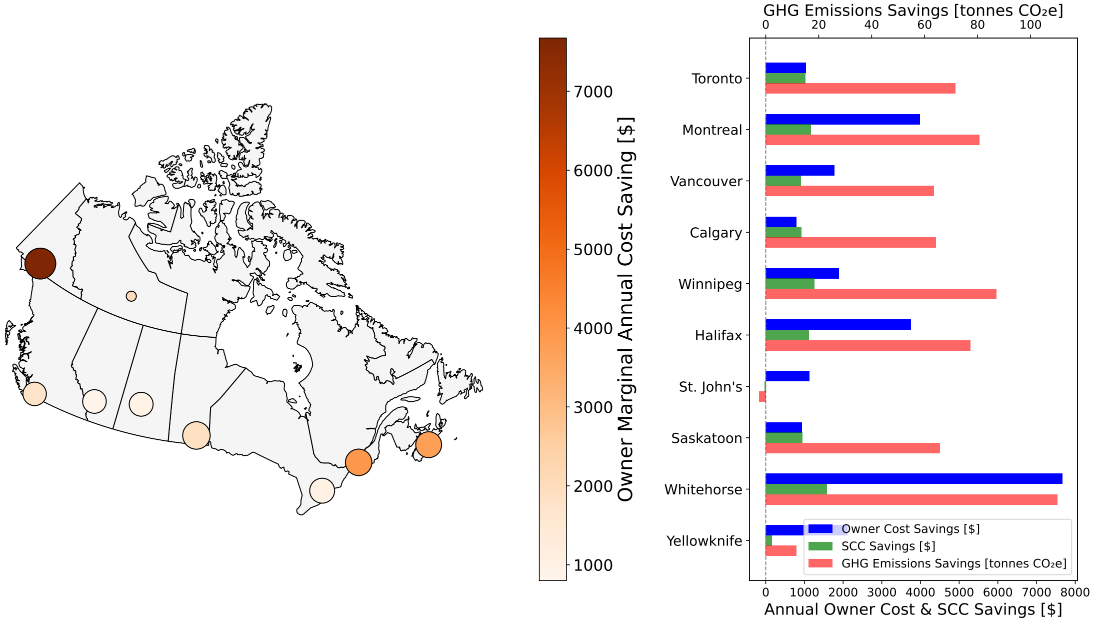

Figure S29. Total GHG and annual cost and SCC saving after building retrofit under 1E5F energy price inflation rates. The panel shows GHG emissions savings (size) and SCC savings (color); map generated using python 3.10 and various libraries: geopandas 1.0.1, matplotlib 3.9.0, and unidecode 1.4.0 (https://www.python.org/).

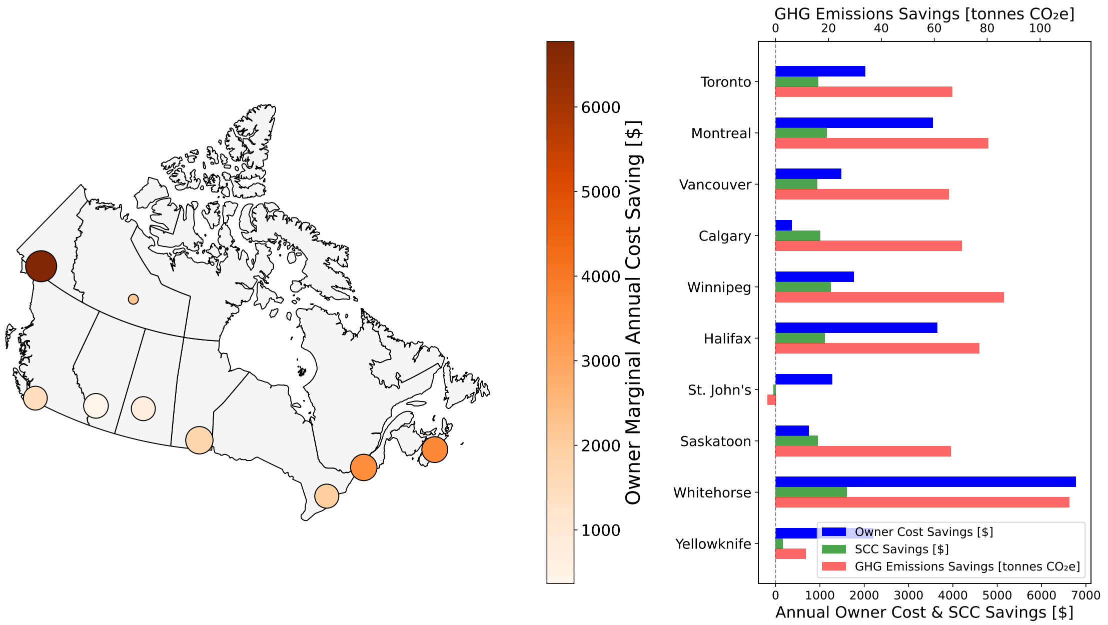

Figure S30. Total GHG and annual cost and SCC saving after building retrofit under 5E5F energy price inflation rates. The panel shows GHG emissions savings (size) and SCC savings (color); map generated using python 3.10 and various libraries: geopandas 1.0.1, matplotlib 3.9.0, and unidecode 1.4.0 (https://www.python.org/).

References

1. Bank of Canada. Bank of canada. https://www.bankofcanada.ca/ (2026). Accessed: 13 January 2026.
